# The Dynamic Landscape of 3′‐UTR Alternative Polyadenylation Across Mouse Fetal Development and Anatomy

**DOI:** 10.1002/advs.202502443

**Published:** 2025-03-24

**Authors:** Qin Wang, Xin Chen, Xiao‐Ou Zhang

**Affiliations:** ^1^ Shanghai Key Laboratory of Maternal and Fetal Medicine Clinical and Translational Research Center of Shanghai First Maternity and Infant Hospital Frontier Science Center for Stem Cell Research School of Life Sciences and Technology Tongji University Shanghai 200092 China

**Keywords:** 3′‐UTR APA, fetal brain development, mouse fetal development, Rbm38, transposable elements

## Abstract

Alternative cleavage and polyadenylation (APA) in the 3′‐untranslated region (3′‐UTR) of mRNA produces transcripts with varied 3′‐UTR and plays a key role in development and organogenesis. This work characterizes 3′‐UTR APA using 85 high‐quality RNA‐seq datasets encompassing 12 tissue types and eight developmental stages of mouse fetuses. Results show that 46.4% of expressed genes undergo APA in a tissue‐specific manner. Changes in polyadenylation site (pAS) usage often operate beyond transcriptional control, revealing APA as an additional layer of gene regulation. Sequence analysis demonstrates that pAS selection, governed by polyadenylation signal strength and adenine preferences, is evolutionarily conserved between mice and humans. Intriguingly, brain tissues display complex 3′‐UTR APA dynamics during development, potentially regulated by RNA‐binding proteins such as *Rbm38*, potentially impacting 3′ UTR extension by restricting distal pAS usage. These APA events are associated with a depletion of conserved miRNA binding sites and an enrichment of transposable elements within alternative 3′ UTRs. To facilitate further research, this work develops APApedia (http://xozhanglab.com/apapedia/), a comprehensive database cataloging identified 3′‐UTR APA events, which serves as a valuable resource for the community to study APA in development and tissue‐specific regulation. This comprehensive resource aids in deciphering the functional implications of APA in mouse fetal development.

## Graphical abstract

This work maps 3′‐UTR APA across mouse fetal development and tissues, identifying conserved sequence determinants that influence pAS choice. In fetal brain tissues, complex APA dynamics during development are potentially regulated by RBPs like *Rbm38*, which restrict distal pAS usage. These APA events correlate with miRNA binding site depletion and transposable element enrichment in alternative 3′‐UTRs.

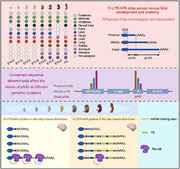

## 1 Introduction

Pre‐mRNA cleavage and polyadenylation is a critical step in mRNA maturation, comprising 3′ cleavage and addition of the polyA tail. Nearly all eukaryotic mRNAs and some non‐coding RNAs require this process for 3′ end formation, which is catalyzed by polyA polymerase and core polyadenylation factors.^[^ ^1^ ^]^ Beyond ensuring transcript stability, polyadenylation dynamically regulates mRNA metabolism, including localization, translation efficiency, and interaction with RNA‐binding proteins (RBPs) and miRNAs.^[^ ^2^ ^]^ Recent advances in high‐throughput sequencing have revealed that over 70% of mammalian genes harbor multiple polyadenylation sites (pAS), which enables alternative polyadenylation (APA) to generate transcript isoforms with distinct 3′ termini.^[^ ^3^, ^4^ ^]^ Among APA subtypes, 3′‐UTR APA—where alternative pAS reside within the 3′ untranslated region (UTR)—stands out as a key regulatory mechanism. By altering 3′ UTR length while preserving coding sequences, 3′‐UTR APA reshapes the cis‐regulatory landscape, thereby fine‐tuning post‐transcriptional control in a context‐dependent manner.^[^ ^3^, ^5^, ^6^ ^]^ To dissect the molecular logic underlying APA regulation, Madeline et al. developed CPA‐Perturb‐seq—a high‐throughput single‐cell platform which enables multiplexed perturbation of 42 cleavage and polyadenylation (CPA) regulators.^[^ ^7^ ^]^ Their work not only delineated how nuclear RNA processing steps orchestrate intronic versus 3′‐UTR APA choices but also decoded a cis‐regulatory code for polyA site usage through deep learning (APARENT‐Perturb). Importantly, 3′‐UTR APA exhibits dynamic spatiotemporal regulation. For instance, brain‐specific isoforms frequently exhibit extended 3′UTRs compared to those in blood or testis,^[^ ^8^, ^9^, ^10^ ^]^ a feature potentially linked to neuron‐specific regulatory complexity. Similarly, during C. elegans embryogenesis, APA patterns are under strong selective pressure, with distinct gene classes exhibiting stage‐specific 3′ UTR dynamics.^[^ ^11^ ^]^ Beyond developmental control, extracellular signals can globally modulate 3′‐UTR APA. A notable example occurs in syncytiotrophoblast differentiation, where coordinated 3′ UTR shortening and intronic APA activation accompany secretory pathway engagement,^[^ ^12^, ^13^ ^]^ highlighting the role of APA as a responsive mechanism bridging environmental cues to post‐transcriptional regulation. Additionally, accumulated lines of evidence highlight the critical role of 3′‐UTR APA in neurodevelopment and neurodegenerative diseases,^[^ ^14^, ^15^ ^]^ noting that longer 3′ UTRs are prevalent in autism and widespread APA changes are observed in amyotrophic lateral sclerosis brains. Furthermore, a recent transcriptome‐wide association study^[^ ^16^ ^]^ underscores the importance of APA in nominating susceptibility genes for brain disorders, often overlooked by traditional analyses. Despite this acknowledged significance, our systematic understanding of the tissue‐specific dynamic patterns of 3′‐UTR APA across different tissues and developmental stages in the mammalian brain, and their impact on tissue‐specific functions, remains limited.

Mice serve as a vital animal model for studying mammalian organogenesis due to their relatively short gestation period of 21 days from fertilization to birth.^[^ ^17^ ^]^ Mouse embryo development begins with the implantation of the blastocyst (E4.0), followed by gastrulation and germ layer formation (E6.5‐E7.5).^[^ ^17^ ^]^ The embryo then proceeds to the early‐somite stages (E8.0‐E8.5), where neural plate and heart tube formation occurs during the transition from gastrulation to organogenesis.^[^ ^18^ ^]^ During mid‐late fetal development (E10.5‐E16.5), embryonic cells proliferate from hundreds of thousands to over ten million, and develop nearly all major organ systems, making this period essential for post‐gastrulation morphogenesis and organ formation.^[^ ^19^ ^]^ As the pluripotency gene network is repressed, transcript diversity increases, and tissue‐level transcriptome profiling using single‐cell RNA‐seq (scRNA‐seq) from E10.5 to birth shows the dominance of neurogenesis and hematopoiesis at both the cellular and gene levels, collectively contributing to over 40% of identified cell types and one‐third of differential gene expression.^[^ ^20^ ^]^ The transcriptome structure undergoes dynamic changes and co‐/post‐transcriptional regulations, such as alternative promoters,^[^ ^21^ ^]^ splicing,^[^ ^22^ ^]^ and polyadenylation,^[^ ^23^ ^]^ coordinating with tissue and organ development. Although a pioneering study has utilized scRNA‐seq data to systematically characterize the dynamics and patterns of 3′‐UTR APA in 38 major cell types across five development stages (E9.5‐E13.5),^[^ ^23^ ^]^ revealing the critical regulatory role of APA during mouse fetal development, the limited gene coverage and APA profiling resolution of scRNA‐seq have impeded a comprehensive understanding of the 3′‐UTR APA landscape across different tissues during mouse fetal development. While the importance of APA in brain development and disease is increasingly recognized,^[^ ^14^ ^]^ a comprehensive understanding of the tissue‐specific and developmental‐stage‐specific regulation of 3′‐UTR APA and its functional roles remains lacking. Therefore, to elucidate the tissue‐specific functionality of specific pASs and their impact on mouse tissue development, it is essential to conduct detailed characterizations of 3′‐UTR APA in diverse tissues and developmental stages.

To bridge this knowledge gap, we systematically profiled the landscapes of 3′‐UTR APA in 85 mouse datasets from continuous mouse fetal developmental stages generated by the ENCODE project,^[^ ^24^ ^]^ and identified several development‐related 3′‐UTR APA patterns which might play an independent role in achieving tissue‐specific functionalities. We also identified several conserved sequence determinants, which can affect the choice of pASs at different genomic locations. Focusing on mouse fetal brain development, we uncovered brain‐specific 3′‐UTR APA patterns, suggesting potential regulation by RBPs, including *Rbm38*, which may control 3′ UTR extension by restricting distal pAS usage during development. Moreover, alternative 3′ UTRs in the developing brain exhibit depletion of conserved miRNA binding sites and enrichment of transposable elements. Taken together, our analyses provided a valuable resource for deciphering the complexity of APA across mouse fetal development and anatomy.

## 2 Results

### 2.1 Profiling of APA across Mouse Fetal Developmental Stages and Tissues

**Figure 1:**
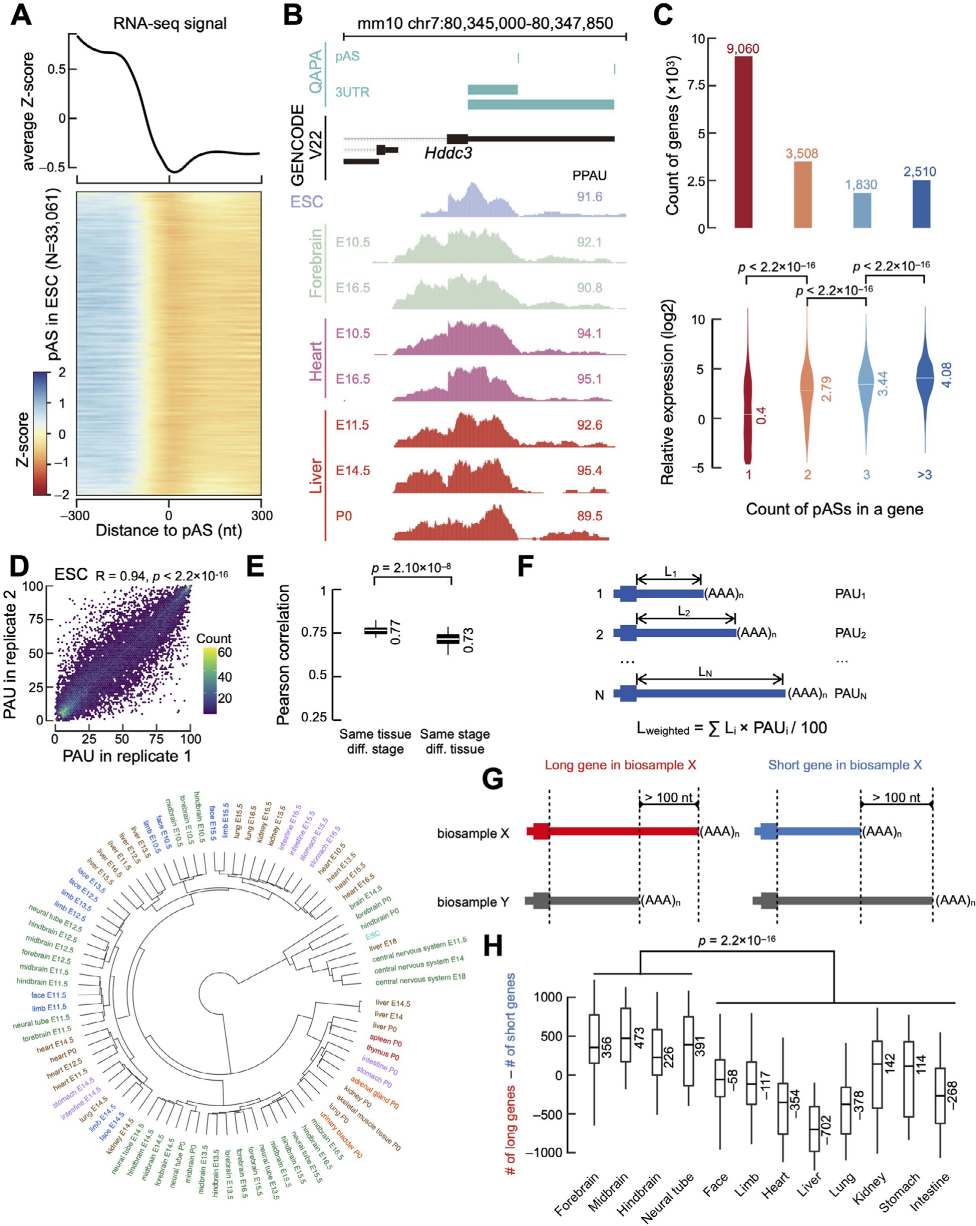
Profiling of APA across mouse fetal developmental time and tissues. A) Normalized RNA‐seq signal (Z‐score) in the ±300‐nt window centered on the 33061 pASs expressed in mESC. B) An example APA event in the gene *Hddc3* in mouse forebrain, heart, and liver tissues. Expressed pASs and 3′ UTR identified by QAPA are shown above GENCODE‐annotated transcripts, and proximal polyA usage (PPAU) values are labeled beside the corresponding RNA‐seq signal track in each sample. C) Number of genes with 1, 2, 3, or >3 expressed pASs across 85 biosamples (top panel). Genes with more expressed pASs have proportionally higher expression levels (bottom panel). Wilcoxon rank‐sum test p‐values are shown. D) Pearson correlation of PAU calculated between biological replicates in mESC is shown along with its p‐value. E) Dendrogram resulting from hierarchical clustering of mouse developmental tissue samples based on their PPAU profiles (bottom panel). Color indicates tissue type. Pearson correlations between samples of the same tissue type at different stages are significantly higher than the correlations across tissues at the same stage, with Wilcoxon rank‐sum test p‐value shown (top‐right panel). F) Definition of weighted 3′ UTR length. Weighted 3′ UTR length for each gene was calculated as the sum of all 3′ UTR lengths of this gene weighted by their corresponding PAUs. N: total number of pASs in a gene. G) Definition of long genes (red) and short genes (blue) in a particular biosample X in comparison with another biosample Y, by comparing the weighted 3′ UTR lengths of the gene between the two biosamples. H) Boxplot showing count differences between long genes and short genes across 12 tissues. Comparisons were made between different tissues at the same developmental stage, as illustrated for forebrain in the Figure S2B, Supporting Information. The Wilcoxon rank‐sum test was performed between brain tissues and non‐brain tissues and the corresponding p‐value is labeled.

We used QAPA^[^ ^25^ ^]^ to identify the expressed pASs from the RNA‐seq data on 85 biosamples generated by the ENCODE project. These biosamples mainly include mouse embryonic stem cells (mESCs) and 12 tissue types spanning developmental stages from embryonic day 10.5 (E10.5) to birth (P0) (Figure S1A,B, Supporting Information; Table S1, Supporting Information). Compared to the previous 3′‐UTR APA profiling study of mouse fetal development,^[^ ^23^ ^]^ which utilized scRNA‐seq data and covered only 8653 genes, we detected 35 243 pASs (66.02% of these pASs are annotated in GENCODE^[^ ^26^ ^]^ and 87.80% of these pASs are in the PolyASite database^[^ ^27^ ^]^) of 16 823 genes expressed in at least one of these 85 biosamples. To evaluate the accuracy of these expressed pASs, we examined the RNA‐seq read density in the ±300 nucleotide (nt) region flanking the pASs in each sample. With mESC as a typical example, the RNA‐seq signal gradually decreases in the 5′‐to‐3′ direction with the lowest signal at each expressed pAS (**Figure** **1**A). To test whether the rise of RNA‐seq signal after the expressed pAS is caused by APA, we separated the pASs in each sample into two groups: the most distal pASs and the remaining pASs. Indeed, when focusing on the most distal pASs, we observed that the gradually decreasing RNA‐seq signal reached its minimum at the pAS and remained flat in the downstream region (Figure S1C, Supporting Information).

To further assess the quality of the identified pASs, we analyzed the sequence motifs and dinucleotide composition in the genomic regions surrounding them. The polyA signal is a hexamer recognized by the cleavage and polyadenylation specificity factors, typically located within 50 nt upstream of the pAS.^[^ ^28^, ^29^ ^]^ Consistent with previous reports,^[^ ^3^, ^4^ ^]^ we found that AAUAAA–the canonical polyA signal—and its variant AUUAAA were the most abundant hexamers, accounting for 38.0% and 13.3% of expressed pASs, respectively (Figure S1D, Supporting Information).

Having generated a catalog of expressed pASs, we next investigated APA across tissues and developmental stages. Notably, among our 35 243 expressed pAS, 33.98% were not annotated in GENCODE (which were from PolyASite instead), and one example is shown in Figure 1B. The proximal pAS of the gene *Hddc3* was used in most tissues, whereas the GENCODE‐annotated pAS was used by only a small fraction of transcripts. To evaluate the prevalence of APA during mouse fetal development, we divided genes into four categories based on the number of expressed pASs (Figure 1C). Among the 16 908 genes expressed (transcripts per million, TPM ≥ 0.1) in at least one of the 85 biosamples, 46.4% possessed more than one expressed pAS across the samples, and genes with more pASs tended to be expressed at higher levels (Figure 1C). These results indicate that APA events occur frequently during mouse fetal development.

We further searched for specific sequence characteristics associated with pASs in each gene category according to the number of expressed pASs (Figure 1C) and found that genes with fewer expressed pASs preferred the strongest polyA signals (AAUAAA) (Figure S1E, Supporting Information; Chi‐square test p‐values < 2.2 × 10^−16^). Accordingly, genes with multiple expressed pASs tended to have adenine at the +1 position, and this nucleotide preference became more striking as the number of pAS increased (Figure S1F, Supporting Information). These results suggest that both the polyA signal sequence and the nucleotide context surrounding a pAS may facilitate the regulation of APA.

To compare APA quantitatively across tissues and developmental stages, we employed the “PolyA Usage” (PAU) metric, which quantifies the relative expression of each pAS with respect to the total expression of all pASs within a gene, and PPAU for the PAU of the proximal pAS (Figure S1A, Supporting Information). PAU showed high reproducibility between biological replicates (Figure 1D and Figure S1G, Supporting Information; Pearson correlation coefficient R = 0.82 ± 0.05 between two biological replicates). In addition, PAU values generated by QAPA were highly consistent with those from PAQR^[^ ^30^ ^]^ and DaPars2,^[^ ^31^ ^]^ two other APA analysis algorithms (Pearson correlation coefficient R = 0.92 with p‐value < 2.2 × 10^−16^ between QAPA and PAQR, and R = 0.94 with p‐value < 2.2 × 10^−16^ between QAPA and DaPars2). Overall, these results indicate that PAU generated by QAPA provides a robust and reliable assessment of pAS usage.

Previous studies reported that 3′ UTRs in mESCs are significantly longer than those in embryonic and adult tissues.^[^ ^32^, ^33^ ^]^ In agreement with these reports, we observed a significant decrease in the PAU of the most proximal pAS, referred to as PPAU, in embryonic and postnatal day 0 stages compared with mESCs (Figure S2A, Supporting Information). Moreover, all tissues exhibit a decrease in PPAU during embryonic development; however, the magnitude and pattern of this change vary among tissues. For example, brain tissues display a consistent and pronounced decrease in PPAU from mESCs to E16.5, whereas liver and stomach exhibit a more gradual reduction. In addition, certain tissues such as kidney and intestine show more marked changes in later developmental stages—specifically from E15.5 to E16.5—while heart and lung maintain relatively stable PPAU levels. Interestingly, some tissues such as face and intestine exhibit a slight rebound in PPAU from E15.5 to E16.5, suggesting a potential decrease in the requirement for 3′ UTR extension during these stages.

Earlier reports have suggested that APA exhibits highly tissue‐specific patterns in mice.^[^ ^5^, ^6^, ^34^ ^]^ To investigate tissue‐specific APA during mouse fetal development, we applied hierarchical clustering to the PPAU profile of all 85 mouse biosamples from 12 tissue types and 8 developmental stages. Notably, the biosamples clustered primarily by tissue rather than by developmental stage (Figure 1E); biosamples from the same tissue but different stages exhibited substantially higher correlations than biosamples from different tissues at the same stage (Figure 1E, median Pearson correlation coefficient 0.77 versus 0.73, p‐value = 2.10 × 10^−8^). These results indicate that the APA profile during mouse development is largely governed by tissue‐specific regulatory programs.

Previous studies have indicated that adult tissues exhibit distinct patterns of 3′ UTR usage.^[^ ^35^, ^36^, ^37^ ^]^ For example, 3′ UTRs in the brain tend to be longer than in testis and ovary, whereas 3′ UTRs in blood are generally shorter, but tissue‐specific patterns of APA during development are hitherto unknown. Therefore, we explored 3′ UTR usage patterns in different tissues during development. We calculated the weighted 3′ UTR length for each gene in a given biosample by summing the lengths of all 3′ UTR isoforms weighted by their corresponding PAUs (Figure 1F). We then compared the weighted 3′ UTR lengths of each gene across tissues and classified a gene as a “long gene” in a tissue if its weighted 3′ UTR length in that tissue was more than 100 nt longer than its weighted 3′ UTR lengths in the other tissues (Figure 1G). Short genes were defined analogously (Figure 1G). Comparing across the 12 fetal tissues, we found that forebrain has a surplus of long genes compared with non‐brain tissues, and this trend became more prominent in later stages, like E15.5 and E16.5 (Figure S2B, Supporting Information). Considering all developmental stages collectively, brain tissues harbored significantly more long genes than non‐brain tissues (Wilcoxon rank‐sum test p‐value < 2.2 × 10^−16^), whereas liver, heart, and lung tissues generally contained more short genes than other tissues (Figure 1H). Together, these results indicate that mouse fetal tissues, similar to adult tissues,^[^ ^3^, ^36^, ^37^, ^38^, ^39^ ^]^ display tissue‐specific preferences for different 3′ UTR lengths, and this specificity becomes increasingly pronounced as development progresses.

### 2.2 Proximal, Middle, and Distal pASs Exhibit Distinct Sequence Characteristics

**Figure 2:**
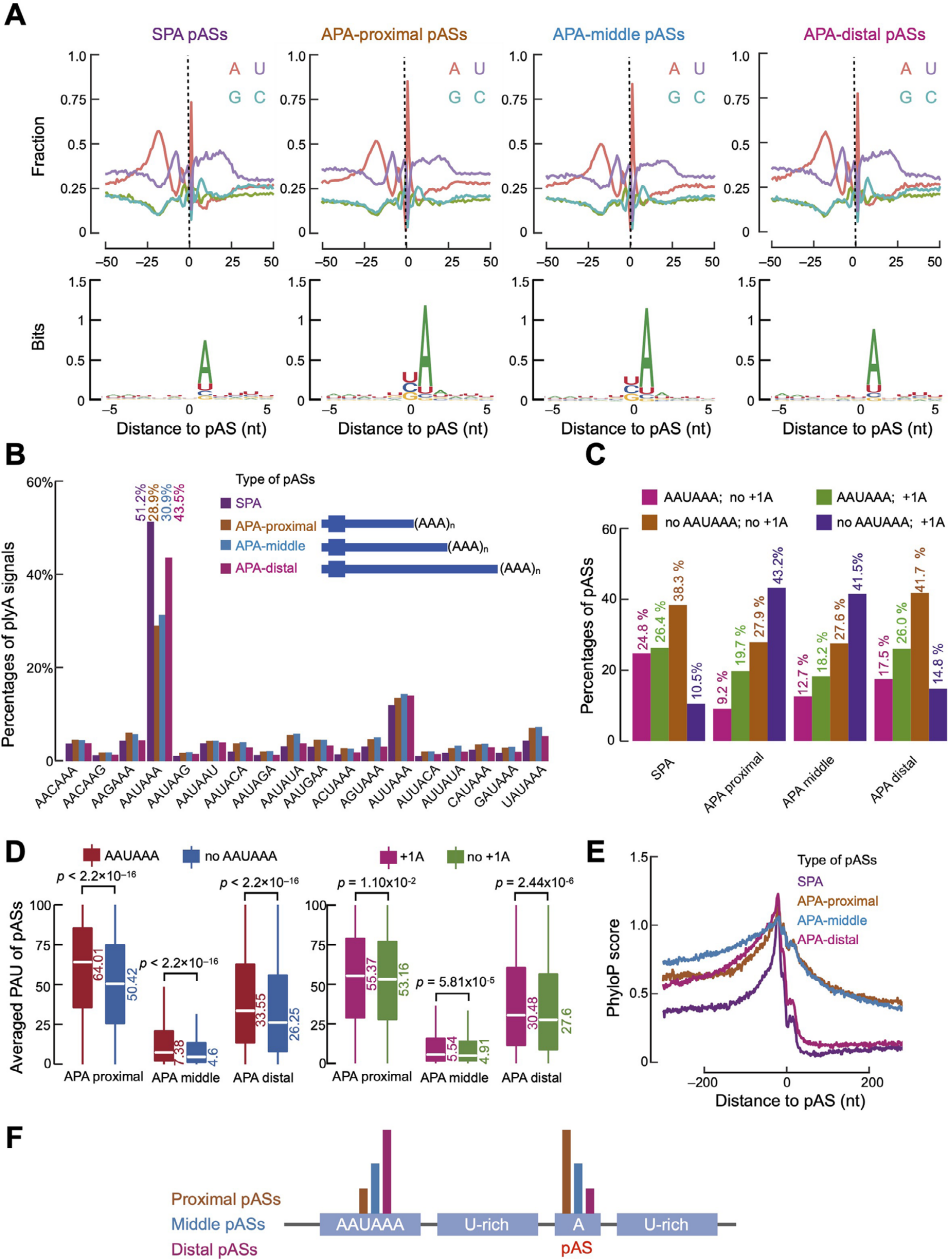
Different types of expressed pASs exhibit distinct sequence characteristics. A) (Top panel) nucleotide composition in the ±50‐nt window centered on mouse SPA pASs, APA‐proximal pASs, APA‐middle pASs, and APA‐distal pASs. (Bottom panel) sequence logos of mouse SPA pASs, APA‐proximal pASs, APA‐middle pASs, and APA‐distal pASs. B) The percentages of 18 polyA signals in the upstream 50‐nt region of mouse SPA pASs (purple), APA‐proximal pASs (yellow), APA‐middle pASs (blue), and APA‐distal pASs (pink). C) Percentages of pASs which have AAUAAA in the upstream 50‐nt region of pASs or adenine at the +1 position for the mouse SPA pASs, APA‐proximal pASs, APA‐middle pASs, and APA‐distal pASs. D) (Left panel) averaged PAU of pASs which have AAUAAA or not in the upstream 50‐nt region for mouse SPA pASs, APA‐proximal pASs, APA‐middle pASs, and APA‐distal pASs. (Right panel) averaged PAU of pASs which have +1A or not for mouse SPA pASs, APA‐proximal pASs, APA‐middle pASs, and APA‐distal pASs. Wilcoxon rank‐sum test was performed between groups and the corresponding p‐values are labeled. E) Averaged phyloP scores of the ±200‐nt genomic regions centered on mouse SPA pASs (purple), APA‐proximal pASs (yellow), APA‐middle pASs (blue), and APA‐distal pASs (pink). F) Schematic diagram to show the regulatory model to choose specific pASs with distinct sequence characteristics. Proximal pASs tend to have weak polyA signals and strong adenine preference at the pAS. In contrast, distal pASs are more likely to have strong polyA signals but weak adenine preference at the pAS.

To further examine the sequence characteristics of pASs in APA genes (i.e., genes with multiple expressed pASs), we defined several types of pASs according to their relative positions in a gene (Figure S1A, Supporting Information): the most proximal pAS (APA‐proximal), middle pASs (APA‐middle; only applicable for genes with three or more expressed pASs), and the most distal pAS (APA‐distal). For comparison, we use single polyA (SPA) to denote genes with just one expressed pAS. We found that APA‐proximal pASs exhibited the highest frequency (0.85) of adenine in the downstream (+1) position; this frequency was lower (0.83, Chi‐square test p‐value = 1.5 × 10^−3^) for APA‐middle pASs and the lowest (0.78, Chi‐square test p‐value = 1.2 × 10^−13^) for APA‐distal pASs (**Figure** **2**A, top panel; note the sharp red peak at distance = 1). Indeed, the sequence logos around the pAS confirmed that the preference for adenine at the +1 position was greatest for the proximal pASs and lowest at the distal sites (Figure 2A, bottom panel). Compared with APA sites, SPA sites still exhibit a high frequency of adenine at the +1 position (Figure 2A). These logos, stratified by pAS type, are consistent with the logos stratified by the total number of pASs (Figure S1F, Supporting Information). Previous studies suggested that nucleotide composition at the pAS alters its binding affinity for the polyadenylation factor *CPSF7*,^[^ ^34^, ^40^ ^]^ contributing to the regulation of APA. The most distal pAS in a gene is usually the strongest presumably to ensure proper transcription termination;^[^ ^3^, ^4^ ^]^ therefore, it is surprising that APA‐distal and SPA pASs show the least frequent +1 A nucleotide at the cleavage site. In addition to the specific adenine preferences directly surrounding the pAS, we observed that pASs of all types were embedded in U‐rich regions as previously reported (Figure 2A, top panel).^[^ ^4^, ^41^ ^]^

In total 18 potential polyA‐signals have been reported,^[^ ^27^ ^]^ although the AAUAAA hexamer is the most frequently observed polyA signal; we examined the usage of these 18 hexamers in the region 50 nt upstream of the pAS for the four types of pASs. The frequency of the AAUAAA hexamer in SPA pASs was 51.2%, whereas the frequencies in APA‐proximal, APA‐middle, and APA‐distal pASs were 28.9%, 30.9%, and 43.5%, respectively (Figure 2B). This pattern is consistent with the notion that the most distal pAS in a gene is the strongest,^[^ ^3^, ^4^ ^]^ but it contrasts with the aforementioned trend for adenine in the pAS +1 position, which decreased for more distal pASs (Figure 2A). We further tested whether the pAS strength and +1 A abundance were correlated, and indeed they were in each type of pASs (Chi‐squared test p‐values < 2.2 × 10^−16^; Figure 2C). Furthermore, the APA‐proximal and APA‐middle groups had much higher percentages of pASs with no AAUAAA but with +1 A (43.2% for APA‐proximal and 41.5% for APA‐middle) than did SPA (10.5%) and APA‐distal (14.8%). In addition, comparing averaged PAUs of pASs with or without AAUAAA or +1 A, we found pASs with AAUAAA or +1 A had higher PAUs than pASs without AAUAAA or +1 A, although the effect of +1 A on elevating PAUs was not as strong as that of AAUAAA (Figure 2D), suggesting that AAUAAA is more important than +1 A for determining the strength of a pAS.

Finally, although pASs show high evolutionary conservation,^[^ ^39^, ^42^ ^]^ the sequences upstream SPA pASs were less conserved than the upstream sequences of all three types of APA pASs (Figure 2E). Furthermore, the sequence conservation of the upstream regions of APA‐middle pASs was higher than that of APA‐proximal and APA‐distal pASs (Figure 2E). This pattern of conservation suggests that genes with multiple pASs might have evolved from an ancestral state that had only one pAS and is consistent with the idea that genes evolve new upstream pASs over time.^[^ ^43^ ^]^

In summary, our results show that polyadenylation is dependent on a specific sequence context and that multiple sequence features, including the presence of adenine at the pAS and the strength of the polyA signal, can regulate pAS choice, leading to diverse APA patterns.

### 2.3 Intrinsic Sequence Characteristics of Expressed pASs are Conserved between Humans and Mice

Our results in mice revealed that pASs located in different parts of the gene exhibit distinct sequence characteristics. Many features of polyadenylation, including cis‐elements, are known to be conserved between mice and humans.^[^ ^4^, ^5^ ^]^ To determine if the sequence features we identified in mice can be generalized to humans, we applied QAPA to the RNA‐seq data from 139 human tissue samples (Table S1, Supporting Information), identifying 42 034 expressed pASs in 16 886 human genes expressed (TPM ≥ 0.1) in at least one of these biosamples. Approximately half of the genes (53.4%) contained more than one pAS, and genes with more expressed pASs tended to be expressed at higher levels (Figure S3A,B, Supporting Information). After classifying human genes based on the number of expressed pASs, we found that the sequence characteristics of the different groups of human pASs were consistent with our results above in mice—namely, the enrichment for specific polyA signals and the nucleotide preferences at the pAS are conserved between the two species (Figure S3C,D, Supporting Information).

Next, we classified human expressed pASs into SPA, APA‐proximal, APA‐middle, and APA‐distal pASs as previously described. Notably, SPA pASs showed the least enrichment for adenine at the +1 position downstream of the pAS (Figure S4A, Supporting Information) and for the strongest polyA signal (AAUAAA) (Figure S4B, Supporting Information). In contrast, in genes with multiple pASs, APA‐proximal pASs exhibited a marked enrichment for adenine at the +1 position and tended to use weaker polyA signals, whereas APA‐distal pASs displayed stronger polyA signals but a lower enrichment for adenine at the pAS (Figure S4A–C, Supporting Information). Similar to mouse pASs, human pASs with AAUAAA or +1 A showed higher PAUs than those without AAUAAA or +1 A, and the impact of AAUAAA on increasing PAUs was stronger than that of +1 A (Figure S4D, Supporting Information). Conservation analysis indicated that SPA pASs were less conserved than APA pASs (Figure S4E, Supporting Information). These results indicate that the sequence characteristics of different types of pASs are similar in humans and mice.

Comparing different types of pASs in mice and humans reveals a conserved regulatory model, demonstrating how polyA signal strength and adenine preferences at the pAS contribute to pAS choice (Figure 2F).

### 2.4 Genes with More Expressed pASs Exhibit Less Tissue Specificity and Tend to be More Highly Expressed

**Figure 3:**
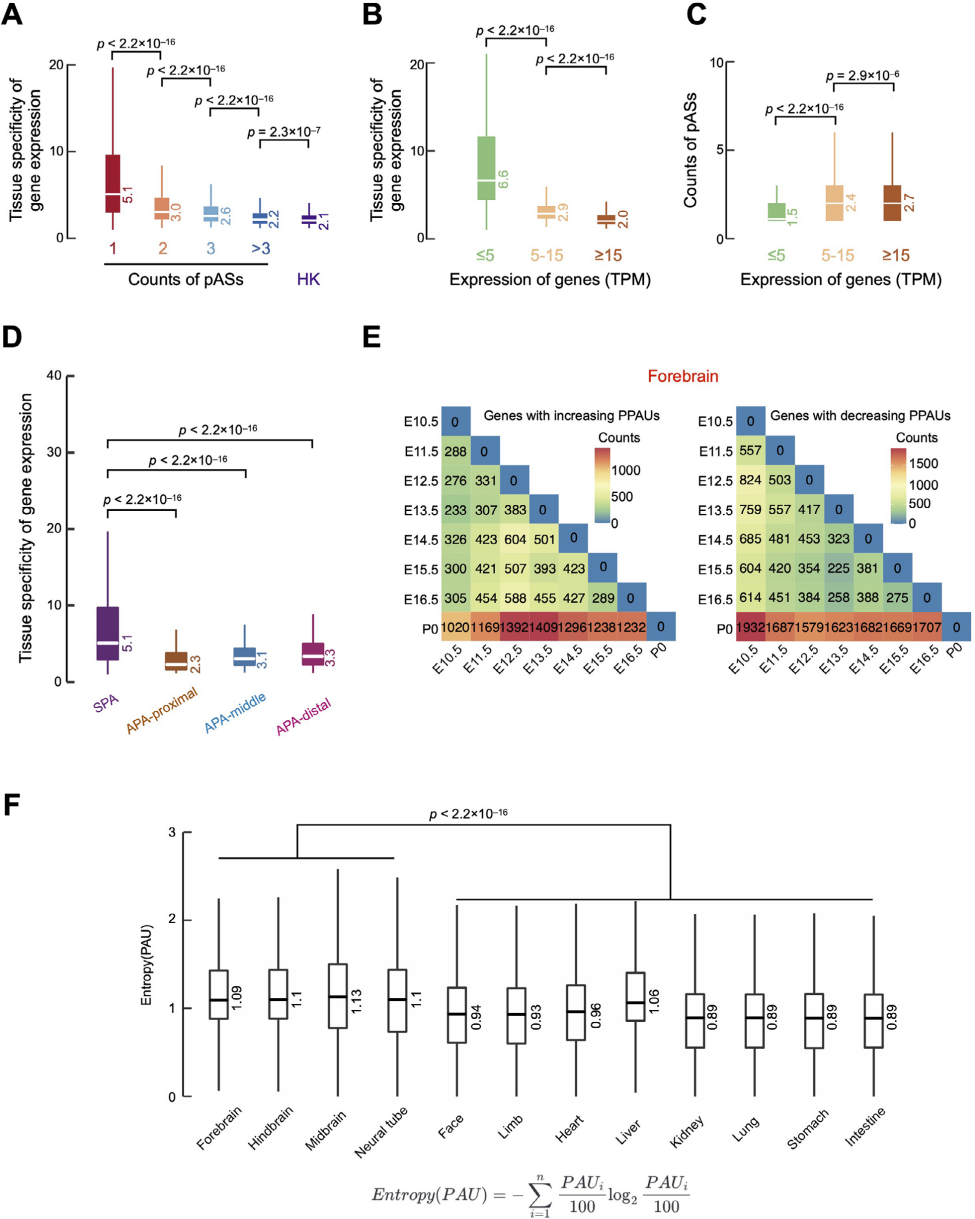
Genes with more expressed pASs exhibit less tissue specificity and tend to be more highly expressed. A) Boxplot showing tissue specificity for genes with different counts of expressed pASs and housekeeping (HK) genes. Wilcoxon rank‐sum test p‐values are shown. B) Boxplot showing tissue specificity for genes with low (TPM ≤ 5), intermediate (5 < TPM < 15), and high (TPM ≥ 15) expression levels. Wilcoxon rank‐sum test p‐values are shown. C) Boxplot showing counts of expressed pASs for genes with low (TPM ≤ 15), intermediate (5 < TPM < 15), and high (TPM ≥ 15) expression levels. Wilcoxon rank‐sum test p‐values are shown. D) Boxplot showing tissue specificity for isoforms corresponding to SPA, APA‐proximal, APA‐middle, and APA‐distal pASs. Wilcoxon rank‐sum test p‐values are shown. E) Counts of genes with increasing (left panel) or decreasing (right panel) PPAUs in the forebrain by comparing between embryonic and postnatal stages. F) The PAU entropy of APA genes in 12 tissues (forebrain, midbrain, hindbrain, neural tube, face, limb, heart, liver, kidney, lung, stomach, and intestine). Wilcoxon rank‐sum test p‐values are shown.

Because genes with different numbers of pASs exhibited distinct sequence characteristics, we examined their enrichment in different gene categories. Using the previously defined tissue specificity score,^[^ ^44^ ^]^ we examined the relationship between tissue‐specific gene expression and the number of expressed pAS. We found that as the number of pASs increased, gene expression became substantially less tissue‐specific; in fact, genes with more than three pASs show low specificity comparable to housekeeping (HK, defined in the HRT Atlas database^[^ ^45^ ^]^) genes (**Figure** **3**A). We then classified genes into three categories based on gene expression: genes with high (TPM ≥ 15), intermediate (5 < TPM < 15), and low (TPM ≤ 5) expression levels. Consistent with previous reports,^[^ ^46^ ^]^ genes with lower expression levels generally had proportionally higher tissue specificity (Figure 3B). Accordingly, highly expressed genes contained more expressed pASs than lowly expressed genes (Figure 3C). Interestingly, APA genes still exhibited lower tissue specificity than SPA genes when we compared the tissue specificity of isoform expression corresponding to APA‐proximal, APA‐middle, APA‐distal, and SPA pASs (Figure 3D). In summary, genes with more pASs exhibit less tissue specificity, tend to be more highly expressed, and are more likely to be housekeeping genes.

### 2.5 APA Events are More Complex in Brain Tissues than in Non‐Brain Tissues during Mouse Fetal Development

To investigate the dynamics of alternative polyadenylation during mouse fetal development, we analyzed the number of genes with significant changes in PPAU (∆PPAU ≥ 20) across 12 tissues from embryonic to postnatal stages. Notably, we observed a significantly lower number of genes with pronounced PPAU alterations between adjacent embryonic stages compared to non‐adjacent stages (Figure 3E; Figure S5, Supporting Information), suggesting that APA changes accumulate progressively throughout development. Furthermore, analysis across tissues showed that four brain regions (forebrain, midbrain, hindbrain, and neural tube) had particularly high levels of PPAU variation, highlighting the unique dynamics of APA in brain development.

Next, although previous studies reported that the regulation of mRNA levels and the change of APA patterns are largely independent in several tissues,^[^ ^47^ ^]^ we sought to determine whether genes that are differentially expressed during mouse fetal development also show significant alterations in PPAU. Differential gene expression analysis of embryonic and postnatal stages revealed many up‐regulated and down‐regulated genes (Table S3, Supporting Information). Strikingly, when comparing these gene expression changes to the changes in PPAU at the same stages, only a small fraction of the differentially expressed genes exhibited notable alterations in PPAU (Figure S6, Supporting Information; Chi‐squared test p‐values in Table S4, Supporting Information). These results suggest that APA provides an independent layer of gene regulation during mouse fetal development.

Given that APA patterns showed substantial changes during mouse fetal development, we wondered whether the complexity of APA events exhibited distinct patterns across different tissues. We defined the entropy of PAU to evaluate the complexity of APA events, and a higher PAU entropy for one gene denoted more APA events occurring in this gene. Comparing across the 12 fetal tissues, we found that four brain tissues (forebrain, midbrain, hindbrain, and neural tube) had significantly higher PAU entropy than other non‐brain tissues (Figure 3F), indicating that APA events in brain tissues are more complex than those in non‐brain tissues and prompting further exploration of APA patterns throughout mouse fetal brain development.

### 2.6 Specific Alternative Polyadenylation Patterns throughout Mouse Fetal Brain Development

**Figure 4:**
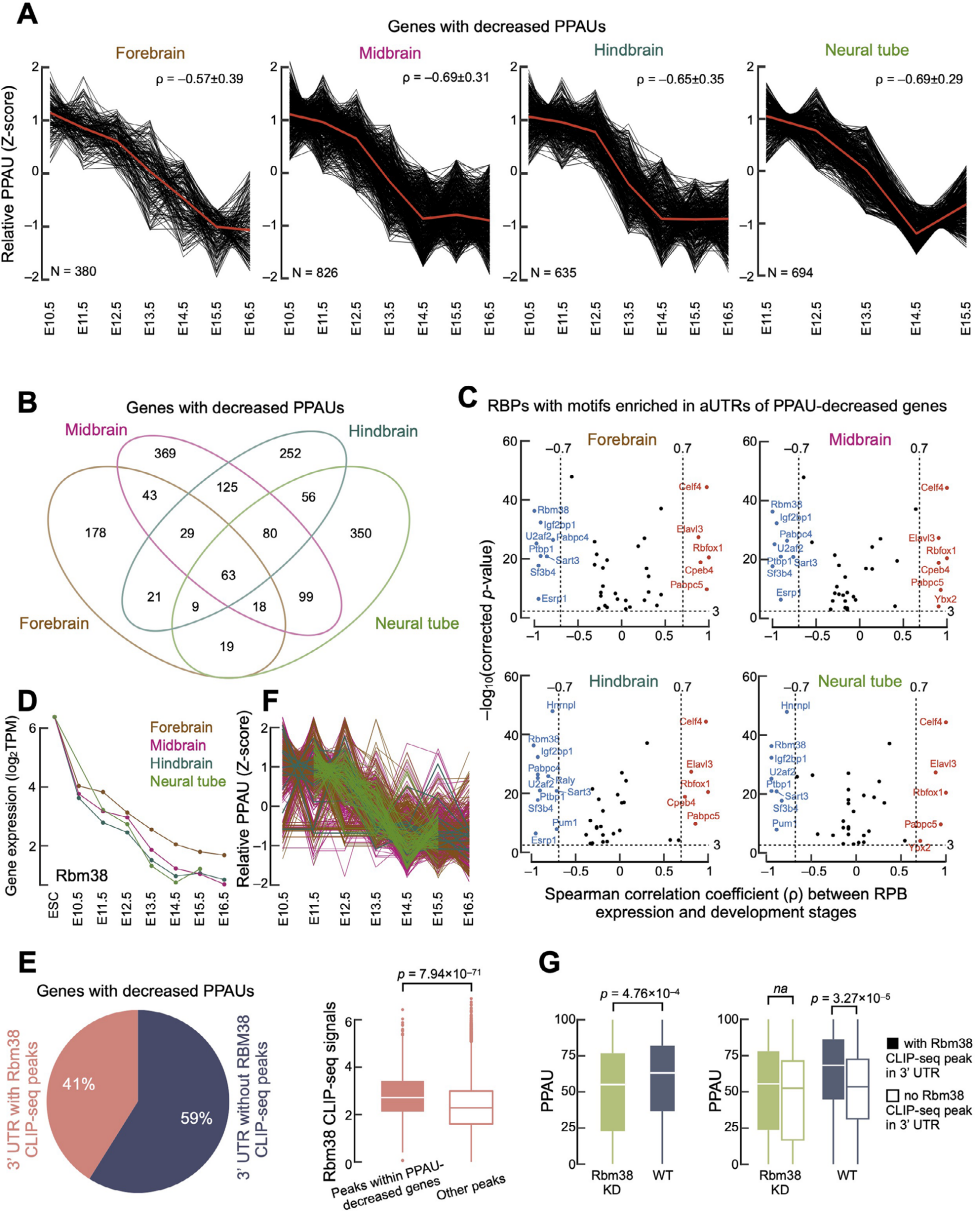
Specific alternative polyadenylation patterns throughout mouse fetal brain development. A) Gene clusters with decreased PPAU pattern (PPAU‐decreased) during mouse fetal development in four brain tissues (forebrain, midbrain, hindbrain, and neural tube). Spearman correlation coefficients ρ were calculated between relative PPAU and development stages for each PPAU‐decreased gene. The median relative PPAUs (Z‐scores) are shown as red lines. B) Venn diagram for genes with PPAU‐decreased patterns identified in four brain tissues (forebrain, midbrain, hindbrain, and neural tube). C) Scatter plots showing RBPs with motifs enriched in aUTRs of PPAU‐decreased genes and their expression trends during mouse fetal brain development. The y‐axis depicts Bonferroni corrected p‐value of RBP motif enrichment in aUTRs over cUTRs. The x‐axis shows Spearman correlation coefficients ρ calculated between TPM of RBPs and development stages. D) Expression trends of *Rbm38* during mouse fetal development in four brain tissues (forebrain, midbrain, hindbrain, and neural tube). E) (Left panel) Distribution of *Rbm38* CLIP‐seq peaks in genes with decreased PPAU patterns. (Right panel) *Rbm38* CLIP‐seq signals in peaks within PPAU‐decreased genes compared to peaks in other genes. Wilcoxon rank‐sum test p‐value is shown. F) Relative PPAU of genes with *Rbm38* CLIP‐seq peaks during mouse fetal development in four brain tissues (forebrain, midbrain, hindbrain, and neural tube). G. Comparison of PPAU values in genes with and without *Rbm38* CLIP‐seq peaks in their 3′ UTRs in wild‐type and *Rbm38* siRNA‐mediated knockdown N2A cells. Wilcoxon rank‐sum test p‐values are shown.

Our results indicate that alternative polyadenylation serves as an independent source of tissue‐specific functionality during mouse development, with more complex APA patterns observed in brain tissues. However, the mechanism driving the tissue‐specific APA pattern in brain tissues remains unclear. A recent study in C. elegans identified four distinct APA patterns during early development, each associated with different functional outcomes.^[^ ^11^ ^]^ To explore the potential functional effects of APA during mouse fetal brain development, we applied clust^[^ ^48^ ^]^ to four mouse embryonic brain samples (from E10.5 to E16.5), identifying clusters in each brain tissue that exhibited a similar PPAU‐decreased pattern (**Figure** **4**A, Spearman correlation coefficient ρ = −0.57 ± 0.39, −0.69 ± 0.31, −0.65 ± 0.35, and −0.69 ± 0.29 for forebrain, midbrain, hindbrain, and neural tube, respectively). Genes in these clusters showed a continuous decrease in PPAU from E10.5 to E16.5 (Figure S7A,B, Supporting Information), and 32.8% (562 out of 1711) of these genes were shared by two or more brain tissues (Figure 4B). Gene ontology analysis revealed enrichment for brain‐related biological processes, such as neurons and projection (Figure S7C, Supporting Information). Furthermore, to explore potential disease relevance, we cross‐referenced these PPAU‐decreased genes with the Human‐Mouse Disease Connection (HMDC) database, which curates experimentally supported orthologous disease‐gene pairs between humans and mice. Strikingly, these genes were enriched for mental health disorders (70 subtypes), including autism spectrum disorder, schizophrenia, and bipolar disorder (Figure S7D, Supporting Information; Table S5, Supporting Information). Notably, the overlapping genes were enriched in synaptic plasticity regulation (GO:00 48167, p = 2.0 × 10^−3^) and included high‐confidence neurodevelopmental risk genes such as *Adnp* (associated with autism and intellectual disability), *Cacna1c* (linked to bipolar disorder and schizophrenia), and *Dyrk1a* (implicated in neuronal proliferation), which are critical for cortical development and synaptic function. These results suggest that APA dynamics during brain development may contribute to psychiatric disorders through isoform‐specific regulation.

A growing number of RBPs are believed to regulate APA during neurogenesis. For example, during fly neurogenesis, ELAV (embryonic‐lethal abnormal visual) proteins inhibit the cleavage and polyadenylation at proximal pASs and recruit paused Pol II to the promoter region, leading to the expression of genes with extended 3′ UTRs.^47,48^ To better understand which RBPs might regulate 3′ UTR extension during mouse fetal brain development, we compared RBP motif matches between the alternative UTRs (aUTRs) and constitutive UTRs (cUTRs) of genes in the PPAU‐decreased clusters and identified 39 RBPs with enriched motifs (Bonferroni corrected p‐value ≤ 1.00 × 10^−3^; Figure 4C). Among them, 10 RBPs (*Rbm38, Elavl3, Celf4, Pabpc5, Rbfox1, Igf2bp1, Ptbp1, Sart3, Sf3b4*, and *U2af2*) exhibited continuously increased or decreased expression during mouse fetal development (Spearman correlation coefficient |ρ| ≥ 0.7; Figure S7E, Supporting Information) and were shared by four brain tissues (Figure S7F, Supporting Information). *Rbm38* is an RBP with a single RNA recognition motif and is associated with multiple functions including the control of mRNA stability, translation, and splicing.^[^ ^49^ ^]^ However, its role in neural APA has not been previously described. Notably, we observed a significant, continuous decrease in *Rbm38* expression during mouse fetal brain development (Figure 4D), and genes with *Rbm38* motifs in their 3′ UTRs displayed a shift from shorter to longer 3′ UTR isoforms as development progressed (Figure S7G, Supporting Information). To further investigate the role of *Rbm38* in the developmental regulation of APA, we examined iCLIP‐seq data for *Rbm38* from the mouse neuroblastoma N2A cell line.^[^ ^49^ ^]^ Among the 1711 genes exhibiting a PPAU‐decreased pattern in mouse fetal brain development, 41% (703) contained significant *Rbm38* iCLIP‐seq peaks in their aUTRs (Figure 4E, left panel), with these peaks showing significantly higher iCLIP‐seq signals compared to other peaks (Figure 4E, right panel; Wilcoxon rank‐sum test p‐value = 7.94 × 10^−71^). These genes exhibited continuous 3′ UTR extension from E10.5 to E16.5 across all four brain regions (Figure 4F). We further examined the 3′‐UTR APA profiles following siRNA‐mediated knockdown of *Rbm38* in N2A cells.^[^ ^49^ ^]^ Knockdown of *Rbm38* led to a significant decrease in PPAU (Figure 4G, left panel). When comparing PPAU between genes with and without *Rbm38* iCLIP‐seq peaks in their 3′ UTRs, we found that genes with *Rbm38* peaks showed significantly lower PPAU in wild‐type N2A cells, but no such difference was observed following *Rbm38* knockdown (Figure 4G, right panel). These findings suggest that *Rbm38* plays a role in preventing 3′ UTR extension by repressing the use of distal pASs.

Taken together, our data suggest that the decrease in *Rbm38* during mouse fetal brain development may contribute to increased usage of distal pAS usage, thereby promoting the extension of 3′ UTRs in mouse fetal brain genes.

### 2.7 Alternative 3′ UTRs Occurring during the Mouse Fetal Brain Development are Depleted of Conserved miRNA Binding Sites but Enriched with Transposable Elements

**Figure 5:**
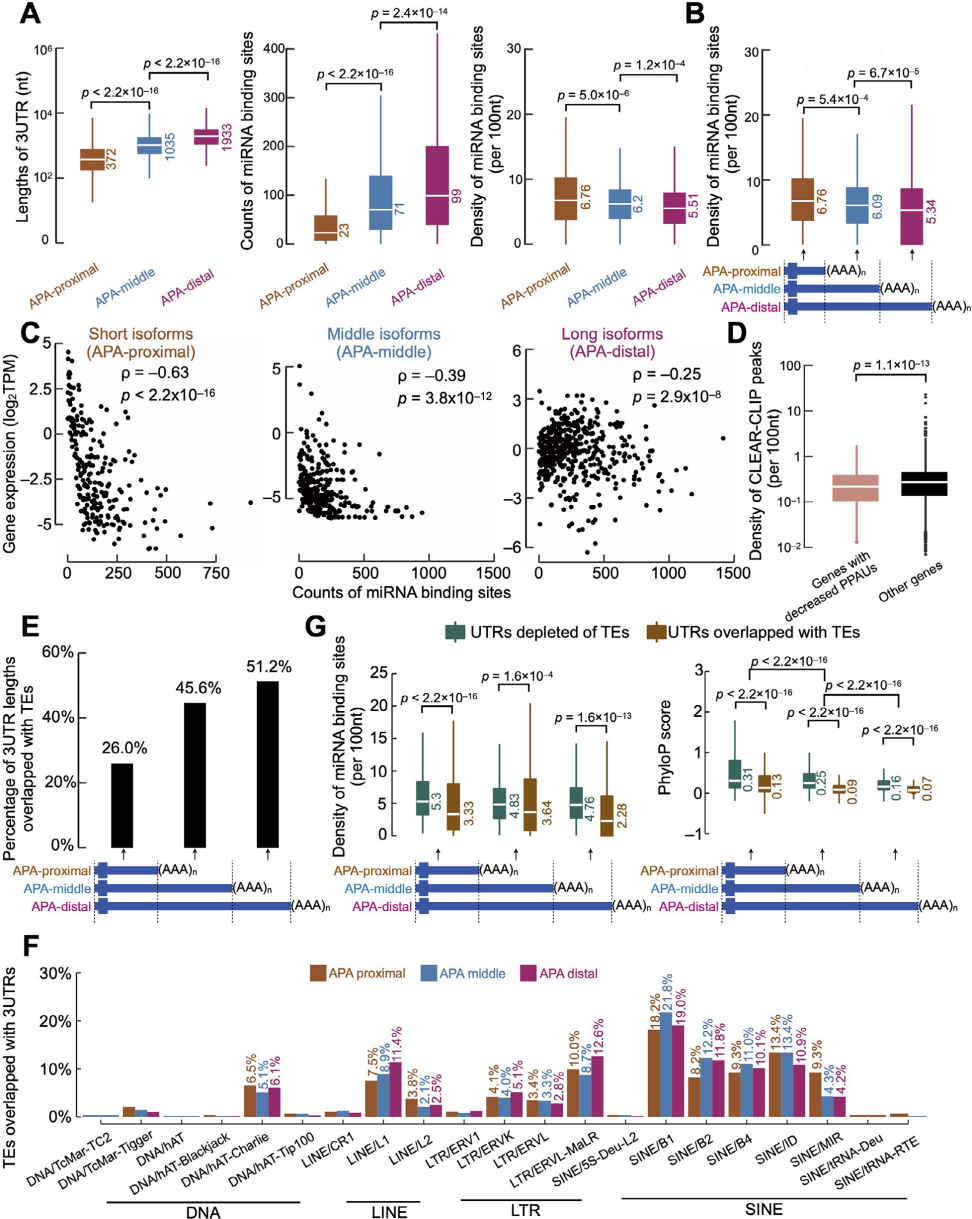
Alternative 3′ UTRs are depleted of conserved miRNA binding sites but enriched with transposable elements. A) The 3′ UTR length (left panel), counts of miRNA binding sites (middle panel), and miRNA binding site density (right panel) in UTRs corresponding to APA‐proximal, APA‐middle, and APA‐distal pASs. Note that APA‐proximal, APA‐middle, and APA‐distal UTRs were used to denote these three types of UTRs, respectively. Wilcoxon rank‐sum test p‐values are shown. B) The miRNA binding site density in specific regions of APA‐proximal, APA‐middle, and APA‐distal UTRs. Wilcoxon rank‐sum test p‐values are shown. Types of UTRs were defined as panel A. C) Correlations between counts of miRNA binding sites and the relative expression of short, middle, and long isoforms. Note that short, middle, and long isoforms correspond to isoforms with APA‐proximal, APA‐middle, and APA‐distal UTRs, respectively. Spearman correlation coefficients and p‐values are shown. Types of UTRs were defined as panel A. D) The CLEAR‐CLIP peak density in 3′ UTRs of PPAU‐decreased genes and other genes. Wilcoxon rank‐sum test p‐value is shown. E) Percentage of 3′ UTR lengths overlapped with transposable elements in the specific regions of APA‐proximal, APA‐middle, and APA‐distal UTRs. Types of UTRs were defined as panel A. F) Density of miRNA binding sites (left panel) and PhyloP scores (right panel) in the specific regions of APA‐proximal, APA‐middle, and APA‐distal UTRs overlapped with or depleted of transposable elements. Wilcoxon rank‐sum test p‐values are shown. Types of UTRs were defined as panel A. G) Percentages of transposable families located in the specific regions of APA‐proximal, APA‐middle, and APA‐distal UTRs. Types of UTRs were defined as panel A.

Accumulated evidence suggests that tissue‐specific APA patterns result in part from the interplay between alternative 3′ UTR isoform expression and the presence of miRNA binding sites in 3′ UTRs.^[^ ^8^, ^33^ ^]^ To test this hypothesis, we divided the 3′ UTRs of all PPAU‐decreased genes identified across four brain tissues into three groups: APA‐proximal, APA‐middle, and APA‐distal UTRs, corresponding to different polyadenylation sites (pASs). We then systematically characterized the distribution of miRNA binding sites in each group. APA‐distal UTRs with the longest length tended to contain most miRNA binding sites, whereas APA‐proximal UTRs contained the fewest miRNA binding sites due to their shortest UTR length (**Figure** **5**A, left and middle panel). However, when normalized to UTR length, APA‐distal and APA‐middle UTRs exhibited a lower miRNA binding site density than APA‐proximal UTRs (Figure 5A, right panel). This pattern was also observed when we compared specific regions within each UTR type (Figure 5B). We hypothesized that the low density of miRNA binding sites in APA‐distal and APA‐middle UTRs might attenuate miRNA‐mediated regulation of long (APA‐distal) and middle (APA‐middle) isoforms. Indeed, we observed a strong negative correlation between the number of miRNA binding sites and expression levels for short (APA‐proximal) isoforms (Figure 5C, left panel; Spearman correlation coefficient ρ = −0.63 with p‐value < 2.2 × 10^−16^), but the correlations for long and middle isoforms were weak (Figure 5C, middle and right panel; Spearman correlation coefficient ρ = −0.25 and −0.39 with p‐values < 2.2 × 10^−16^ for long and middle isoforms, respectively). To further experimentally validate the hypothesized impact of miRNA binding on APA isoform expression, we analyzed CLEAR (covalent ligation of endogenous Argonaute‐bound RNAs)‐CLIP data from mouse cortex. Consistent with our hypothesis, genes exhibiting decreased PPAU in mouse fetal brain tissues showed significantly reduced CLEAR‐CLIP peak density (Figure 5D; Wilcoxon rank‐sum test p‐value = 1.1 × 10^−13^).

Reports suggest that 3′ UTRs are hot spots for genomic integration of transposable elements (TEs), such as SINEs.^[^ ^50^, ^51^ ^]^ Embedded TEs may contribute to regulatory processes, including nuclear export, translation, and mRNA decay.^[^ ^50^, ^51^ ^]^ To investigate whether transposable elements are differentially distributed across alternative UTRs, we analyzed the overlap between TEs and APA‐proximal, APA‐middle, and APA‐distal UTRs. We found that APA‐distal and APA‐middle UTRs were proportionally more enriched in TEs than APA‐proximal UTRs (Figure 5E; 51.2% and 45.6% versus 26.0%, Chi‐squared test p‐values < 2.2 × 10^−16^). Moreover, while APA‐proximal UTRs were preferentially integrated by ancient TE families such as MIR^[^ ^52^ ^]^ and L2,^[^ ^53^ ^]^ APA‐distal and APA‐middle UTRs were enriched for rodent‐specific TE families, including B1 and B2^[^ ^54^ ^]^ (Figure 5F, Chi‐squared test p‐values < 2.2 × 10−16). We reasoned that the presence of species‐specific TEs, which contain few conserved miRNA binding sites, might contribute to the depletion of miRNA binding sites in APA‐distal and APA‐middle UTRs. Consistent with this hypothesis, UTRs containing TEs exhibited a significantly lower miRNA binding site density than UTRs lacking TEs, with the effect being most pronounced in APA‐proximal UTRs (Figure 5G, left panel). Accordingly, UTRs containing TEs showed lower evolutionary conservation than TE‐depleted UTRs, and APA‐distal and APA‐middle UTRs were less conserved than APA‐proximal UTRs (Figure 5G, right panel). Together, these findings suggest that the presence of low‐conserved transposable elements in alternative UTRs contributes to the depletion of miRNA binding sites, thereby reducing miRNA‐mediated regulation of long isoforms during mouse fetal brain development.

### 2.8 APApedia Provides Efficient Interface for APA Search and Visualization across Mouse and Human Developmental Tissues

To facilitate the search and visualization of APA landscapes across mouse and human development, we developed an integrative database called APApedia (http://xozhanglab.com/apapedia/). This database contains APA events from all 257 developmental samples, including 13 developmental stages and 35 tissue types in mice, and 28 developmental stages and 41 tissue types in humans. APA events from a broader spectrum of samples will be added as additional high‐quality RNA‐seq datasets are available.

Using this online database, users can easily query a gene of interest using a gene symbol or Ensembl ID. APApedia will retrieve all of the pASs for this gene, related miRNA binding sites, and RBP motif sites in corresponding cUTRs and aUTRs (Figure S8A, Supporting Information). In addition, users can retrieve and visualize APA patterns for available developmental stages and tissues in mice (Figure S8B, Supporting Information). Polyadenylation sites, APA events, miRNA binding sites, and RBP motifs can also be visualized in the website‐embedded JBrowse (Figure S8C, Supporting Information).^[^ ^55^ ^]^ Different types of tracks, including gene annotation from GENCODE, 3′ UTRs, pASs, and RNA‐seq tracks from different tissue types, are available in JBrowse. Finally, users can access tables for pASs and APA events for each sample from the downloads page.

APApedia is a useful and user‐friendly resource for retrieving and visualizing APA events across developmental tissues, designed to explore the latent regulatory mechanisms of APA during mouse fetal development.

## 3 Discussion

Alternative cleavage and polyadenylation is a conserved process that generates multiple mRNA isoforms with different 3′ ends. This RNA regulatory mechanism exists in all eukaryotes and is associated with crucial biological processes like development and organogenesis.^[^ ^3^, ^5^, ^6^ ^]^ In this study, we performed a comprehensive analysis of APA events using 85 mouse RNA‐seq biosamples spanning 12 tissue types and 8 developmental stages, identifying 35 243 expressed pASs—representing 10.6% of the annotated pASs in the mouse genome—in at least one biosample. Overall, 46% of expressed genes underwent APA during mouse fetal development (Figure 1). Notably, sequence analysis revealed that several sequence determinants governing pAS selection—polyadenylation signal strength and adenine preferences—are conserved between mice and humans (Figure 2; Figures S3 and S4, Supporting Information), suggesting evolutionary conservation of APA regulatory logic. These findings provide a foundation for extending insights into human development and disease contexts, further supported by the cross‐species query modules in the APApedia database.

Earlier analyses of APA in adult mouse tissues^[^ ^5^, ^6^, ^34^ ^]^ showed that different tissues produced mRNAs with different 3′ UTR lengths and that APA was highly tissue‐specific. Our results are consistent with these findings and further show that tissue specificity of APA increases throughout mouse fetal development (Figures 1 and 3). This dynamic regulation likely enables precise control of gene expression during organogenesis. For instance, the progressive extension of 3′ UTRs in brain tissues (Figure 4) may facilitate interactions with neuron‐specific RBPs, reflecting an increasing complexity in APA regulation. Notably, although APA events are tissue‐specific at all developmental stages (Figure 1), their specificity becomes more pronounced as fetal development progresses (Figure S5, Supporting Information). Moreover, APA landscapes exhibit gradual shifts across developmental stages, with significant changes emerging in later stages (Figure 3; Figure S5, Supporting Information), suggesting a cumulative process underlying tissue‐specific APA regulation.

The observed depletion of conserved miRNA binding sites in alternative 3′ UTRs (Figure 5) highlights a critical functional consequence of APA. Typically, miRNAs bind to conserved sites in 3′ UTRs to mediate mRNA degradation or translational repression. By selectively removing these evolutionarily constrained miRNA targets, APA may fine‐tune post‐transcriptional regulation, particularly for genes involved in key developmental pathways. For example, genes with extended 3′ UTRs in the fetal brain are enriched for neurodevelopmental disorders such as autism spectrum disorder and schizophrenia, suggesting that APA‐mediated miRNA site depletion could fine‐tune neuronal gene expression during critical developmental windows. Given that over 70% of protein‐coding genes in both human and mouse genomes employ APA to generate mRNA isoforms with different 3′ UTRs,^[^ ^3^, ^4^ ^]^ shifts in the relative abundance of these isoforms represent an additional layer of gene regulation, enabling tissue‐specific functionality.^[^ ^47^ ^]^ Our results further reveal that genes with multiple active pASs tend to exhibit higher expression levels and lower variability across samples than genes with only one or few expressed pASs (Figure 3). Furthermore, our stage‐specific APA profiling identified numerous genes with significant APA alterations, many of which participate in organ morphogenesis (Figures S5 and S6, Supporting Information), reinforcing the contribution of APA to developmental programs.

Echoing earlier studies,^[^ ^5^, ^56^ ^]^ we observed that distinct sequence motifs are associated with pASs at different positions (Figure 2; Figures S3 and S4, Supporting Information). We propose a regulatory model in which the choice of pAS in APA genes depends on the interplay between polyadenylation signal strength and adenine preference (Figure 2F). Proximal pASs generally exhibit weaker polyadenylation signals but higher adenine preference, whereas distal pASs tend to have stronger signals but lower adenine preference, highlighting distinct regulatory influences on pAS selection. This quantitative model of pAS selection, validating the interplay of signal strength and adenine preference, is a novel contribution to the understanding of APA regulation. Although polyadenylation signals have a dominant effect, our data indicate that proximal pASs show higher PAUs than distal pASs (Figure 2D; Figure S4D, Supporting Information). This apparent discrepancy may be explained by a “first come, first served” mechanism in proliferating cells,^[^ ^57^ ^]^ where proximal pASs are transcribed earlier, providing more time for recognition and processing by polyadenylation factors. Further investigation is needed to clarify the interplay between these cis‐elements in APA regulation.

Brain tissues are characterized by more intricate co‐/post‐transcriptional regulation compared to other tissues, particularly in the context of non‐canonical alternative splicing.^[^ ^58^, ^59^, ^60^ ^]^ Our results indicate that APA events are more complex in brain tissues than in non‐brain tissues (Figure 3), with continuous 3′ UTR extension observed during mouse fetal brain development (Figure 4). Importantly, genes with extended 3′ UTRs in the brain are significantly enriched for neurodevelopmental and psychiatric disorders (e.g., autism spectrum disorder and schizophrenia). This aligns with growing evidence that APA generates mRNA isoforms with distinct roles in neuronal differentiation and synaptic plasticity.^[^ ^14^ ^]^ Our APA atlas may serve as a valuable resource to prioritize disease‐relevant polyA sites for functional studies or therapeutic intervention. Although it remains challenging to differentiate whether these APA patterns arise from differential pAS selection or from post‐transcriptional miRNA‐mediated degradation, our datasets suggest that both mechanisms contribute. We identified several RBPs that interact with alternative UTRs, potentially modulating pAS usage. For example, *Rbm38* appears to suppress distal pAS usage in neural tissues; however, its expression decreases during mouse fetal brain development, leading to 3′ UTR extension (Figure 4). The identification of *Rbm38* as a novel regulator of APA, specifically repressing distal pAS usage, represents a significant advance in understanding APA regulation in neural tissues. Moreover, the enrichment of low‐conserved transposable elements coupled with the depletion of conserved miRNA binding sites in alternative UTRs may further reduce miRNA‐mediated repression of longer isoforms (Figure 5). Additionally, TE sequences themselves may introduce novel regulatory elements, including binding motifs for RBPs that promote distinct RNA localization patterns or translation efficiencies, independent of miRNA regulation. These findings provide new mechanistic insights into how TE insertions can actively shape APA landscapes and modulate miRNA‐mediated gene regulation.

To empower the broader research community, we developed the APApedia database. This resource catalogs all identified APA events along with tissue‐ and stage‐specific PAU values, conserved sequence features, and annotations for RBP/miRNA binding sites. By supporting cross‐species comparisons and disease‐oriented queries, APApedia facilitates rapid identification of APA events with potential relevance to human development and pathology. This database bridges mechanistic insights from model organisms to clinical relevance, facilitating hypothesis generation regarding the role of APA in disease etiology and therapeutic targeting.

In summary, we provide a comprehensive characterization of APA events across mouse fetal development and anatomy, offering a rich and reliable resource for the APA research community. Our study reveals a dynamic APA landscape with distinct genomic features that underpin tissue‐specific and developmental regulation. By integrating these findings into APApedia, we facilitate translational research and hypothesis‐driven investigations into mechanistic and clinical implications of APA. To further solidify the conceptual advances of this study, future studies should focus on cross‐species validation, deeper mechanistic dissection of APA regulation, and the integration of multi‐omics data to fully elucidate the role of APA in development and disease.

## 4 Experimental Section

### Profiling of APA across Mouse Fetal Developmental Time and Tissues

This work analyzed APA dynamics using the QAPA (version: 1.3.0)^[^ ^25^ ^]^ pipeline on ribo– or polyA+ RNA‐seq datasets from the ENCODE Consortium (Table S1, Supporting Information). To ensure technical robustness, samples were first filtered by sequencing depth and adjusted for batch effects using the limma package (v3.42.2).^[^ ^61^ ^]^ Raw reads underwent adapter trimming and quality filtering using Trim Galore. Hierarchical clustering analysis further confirmed that samples clustered according to biological features (tissue and developmental stage) rather than technical variables (Figure S1B, Supporting Information). The pAS annotations were constructed based on the PolyASite database (N = 30 1001) and pASs downloaded from GENCODE Basic gene annotations (mouse: mm10 vM22, N = 31 367; human: hg38 v31, N = 49 884), the corresponding 3′ UTR sequences were then extracted for isoform quantification using the Salmon^[^ ^62^ ^]^ algorithm (version 0.14.0) for each biosample, and the relative usage of each pAS in a gene was summarized using QAPA. Reads from biological replicates were pooled only after confirming high intra‐replicate consistency. A pAS was considered expressed in our study if its corresponding 3′ UTR sequence was detected in at least one biosample. To systematically evaluate APA events, the metric “PolyA Usage” (PAU),^[^ ^25^ ^]^ defined as the ratio of the expression of the corresponding 3′ UTR to the total 3′ UTR expression of the same gene, was calculated for each pAS, and the PAU of the most proximal pAS deemed PPAU (Proximal PAU; Figure S1A, Supporting Information; Table S2, Supporting Information). To exclude potential false positives, PAUs were only calculated for genes with sufficiently high expression levels (TPM ≥ 0.1).

### Characterization of Sequence Features of Active pASs

Genomic coordinates of expressed pASs were directly fetched from the GENCODE basic polyA annotation and the PolyAsite database (pAS annotations with score ≥ 4). If a pAS was annotated by both GENCODE and the PolyAsite database, the genomic coordinates fetched from the PolyAsite database were retained. Expressed pASs were divided into groups according to the genomic location and the count of pASs in a gene, respectively. The frequency of 18 hexamers corresponding to potential polyA signals^[^ ^27^ ^]^ around different groups of pASs was plotted. The sequence logo for each pAS group was generated with ggseqlogo^[^ ^63^ ^]^ (version: 0.1). PhyloP scores of the mouse genome (mm10.60way.phyloP60way.bw) and the human genome (hg38.phyloP100way.bw) were downloaded from UCSC,^[^ ^64^ ^]^ and the corresponding PhyloP scores^[^ ^65^ ^]^ of the genomic positions in a specific region were averaged (excluding protein‐coding positions from the averaging) to quantify the evolutionary conservation of that region.

### Gene Differential Expression and Tissue‐Specificity Analysis

This work downloaded gene expression levels of mouse biosamples (Table S3, Supporting Information) from the ENCODE Portal; these data were generated by the ENCODE uniform processing pipelines for corresponding mouse RNA‐seq datasets. Differentially expressed genes were identified using tximport^[^ ^66^ ^]^ (version: 1.14.2) and edgeR^[^ ^67^ ^]^ (version: 3.28.1) with log2 fold change ≥ 2 for up‐regulated genes or fold change ≤ 0.05 for down‐regulated genes and FDR < 0.01.

We used previously defined tissue‐specific score (TS)^[^ ^44^ ^]^ to evaluate the tissue specificity of gene expression, defined as:

(1) $$\mathrm{TS}=\frac{Max\left(TPM\right)}{Mean\left(TPM\right)}$$

where Max(TPM) and Mean(TPM) represent the highest and average expression of a gene across all tissue biosamples, respectively. A higher TS indicates a higher degree of tissue specificity for gene expression. In order to compare the tissue specificity between different types of genes, we got ≈3000 mouse housekeeping genes from the HRT Atlas database.^[^ ^45^ ^]^

### Analysis for RNA Binding Proteins

AME algorithm^[^ ^68^ ^]^ from the MEME toolkit was used to scan RBP motifs in the MEME CIS‐BP database^[^ ^69^ ^]^ by comparing motif occurrences between the aUTR and cUTR regions of genes in the PPAU‐down groups. Enriched RBP motifs were identified with Bonferroni corrected p‐value ≤ 1.00 × 10^−3^. To check the expression trend of these RBPs during mouse fetal development, we computed the Spearman correlation coefficients between TPM values and development stages for each RBP, and then selected co‐expressed RBP with |ρ| ≥ 0.7. *Rbm38* CLIP‐seq peaks were processed as previously described^[^ ^49^ ^]^ and selected based on an adjusted p‐value < 0.05 and a log2 fold change of iCLIP reads over input reads greater than 0.

PAU entropy to evaluate the complex of APA patterns: We defined the entropy of PAU for APA genes as:

(2) $$\mathrm{Entropy}(\mathrm{PAU})=-\sum_{i=1}^{n}\frac{PAU_{i}}{100}log_{2}\frac{PAU_{i}}{100}$$

The higher the entropy of PAU is, the more complex APA events are. The PAU entropy for one tissue was computed by averaging the PAU entropy calculated in this tissue across all the development stages.

### APA Pattern Recognition throughout the Mouse Fetal Development

This work clustered PPAU profiles throughout the mouse fetal development in forebrain samples using the time series pattern extraction method clust^[^ ^48^ ^]^ (parameter: ‐n 0 3 4) which is based on k‐means clustering, and significant APA patterns were selected for downstream analyses.

### Enrichment Analysis for Gene Ontology, Transposons, and MicroRNAs

Gene ontology enrichment analysis was performed with the gprofiler2^[^ ^70^ ^]^ R package (version: 0.1.9), and enriched gene ontology terms (FDR < 0.01) were extracted. Annotations of transposable elements were downloaded from the UCSC RepeatMasker database.^[^ ^64^ ^]^ High‐confidence binding sites of microRNAs (score ≥ 50) were extracted from the miRDB database.^[^ ^71^ ^]^ Counts of transposons and microRNA binding sites located in the 3′ UTR region of genes were calculated using bedtools.^[^ ^72^ ^]^ The expression levels of short, middle, and long isoforms for 1711 PPAU‐decreased genes were computed by selecting the median TPM from four brain tissues (forebrain, hindbrain, midbrain, and neural tube), and Spearman correlation coefficients were then calculated between the isoform expression and the counts of miRNA binding sites on the corresponding 3′ UTR regions. CLEAR‐CLIP peaks from mouse cortex were downloaded from the Gene Expression Omnibus with accession number GSE73058.

### Implementation of APApedia Database

APApedia is implemented with Django (version: 1.8), Bootstrap, jQuery, Vue.js, and JBrowse. SQLite3 (version: 3.36.0) is used to store metadata information. APApedia is hosted on a CentOS 7 Linux system with Apache HTTP Server (version: 2.4.6) to provide stable web access.

### Statistical Analysis

Statistical analysis was performed using R 4.3.3. The differences between two groups were compared using Wilcoxon rank‐sum test. *p* < 0.05 was considered statistically significant and asterisks denoted statistical significance (*p < 0.05, **p < 0.01, ***p < 0.001).

## Conflict of Interest

The authors declare no conflict of interest.

## Author Contributions

Q.W. and X.C. contributed equally to this work. X.Z. and Q.W. designed the study. X.Z. supervised the study. Q.W. and X.C. performed most of the bioinformatic analyses. X.Z., Q.W., and X.C. wrote the manuscript draft. X.Z. confirmed the final manuscript.

## Supporting information



Supporting Information

Supplemental Table 1

Supplemental Table 2

Supplemental Table 3

Supplemental Table 4

Supplemental Table 5

## Data Availability

The data that support the findings of this study are openly available in ENCODE at www.encodeproject.org/, reference number 24.
