# Supplementary material for: The Dynamic Landscape of 3′‐UTR Alternative Polyadenylation Across Mouse Fetal Development and Anatomy
Source: Adv Sci (Weinh). 2025 Mar 24;12(19):2502443. doi: 10.1002/advs.202502443 (PMC12097126; doi:10.1002/advs.202502443)
Supplement: Supplementary file 1 — Supporting Information [file ADVS-12-2502443-s005.docx]

Supporting Information

**The dynamic landscape of 3′-UTR alternative polyadenylation across mouse fetal development and anatomy**

*Qin Wang, Xin Chen, Xiao-Ou Zhang**

*
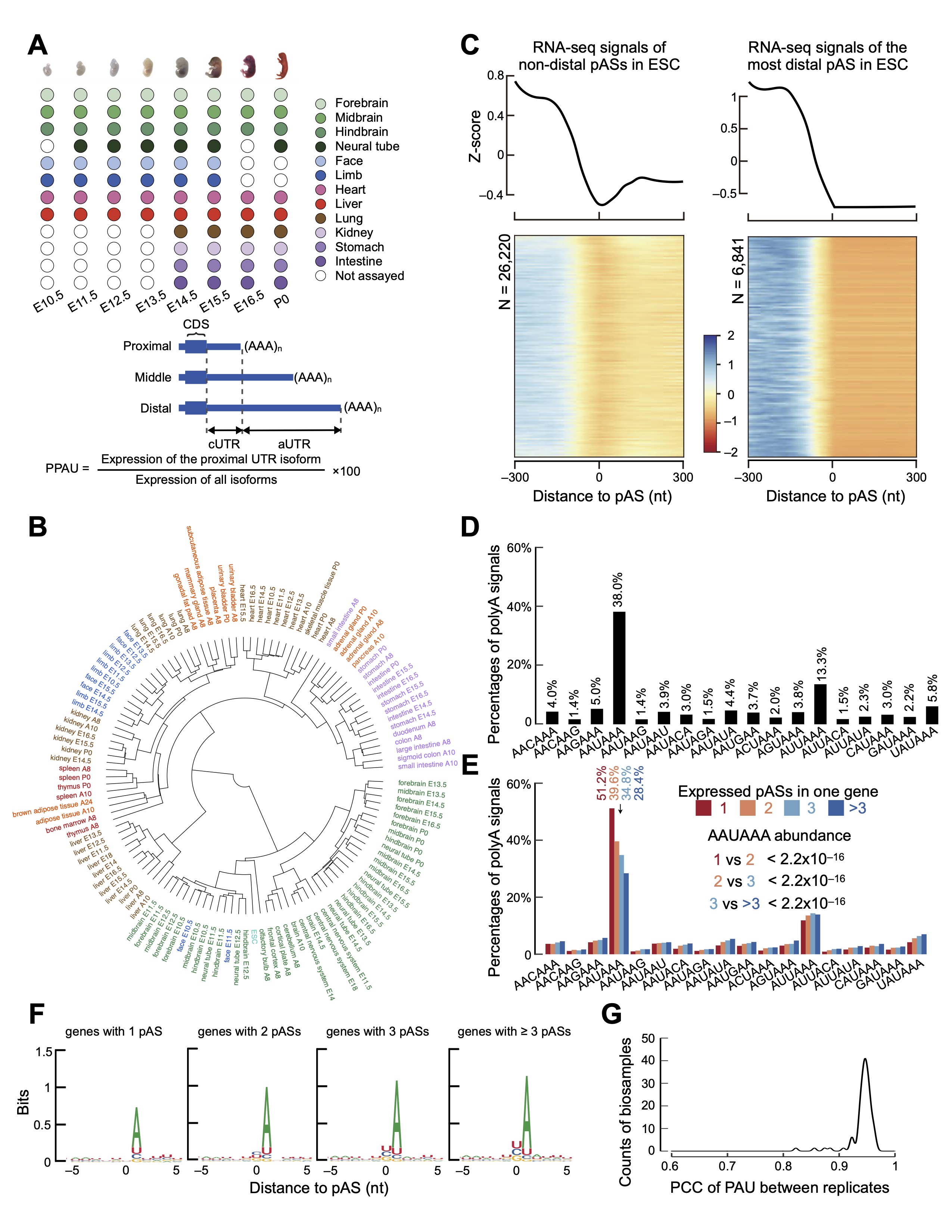
*

**Figure S1. Genome-wide identification of expressed pASs across 85 mouse developmental RNA-seq biosamples**

A) RNA-seq data from 12 tissue types at 4–8 developmental stages were analyzed in this study (top panel). Proximal polyA usage (PPAU) was calculated as the ratio between the expression level of the proximal 3′ UTR and the expression level of all 3′ UTRs (bottom panel).

B) Dendrogram resulting from hierarchical clustering of mouse developmental tissue samples based on their gene expression profiles.

C) Normalized RNA-seq signal (Z-score) in the ±300-nt window centered on the non-distal pASs (left panel) and the most distal pASs (right panel) in mESC.

D) The percentages of 18 polyA signals in the upstream 50-nt region of expressed pASs.

E) The percentages of 18 polyA signals in the upstream 50-nt region of expressed pASs in different gene categories. As defined in Figure 1C, expressed genes were classified into four categories: genes with one pAS (red), two pASs (yellow), three pASs (cyan), and more than three pASs (blue). Chi-square p-values for comparing groups of genes are shown.

F) Sequence logos of expressed pASs in different gene categories. Gene categories were defined as Figure 1C.

G) The Pearson correlation coefficients (PCC) between biological replicates across mouse biosamples.


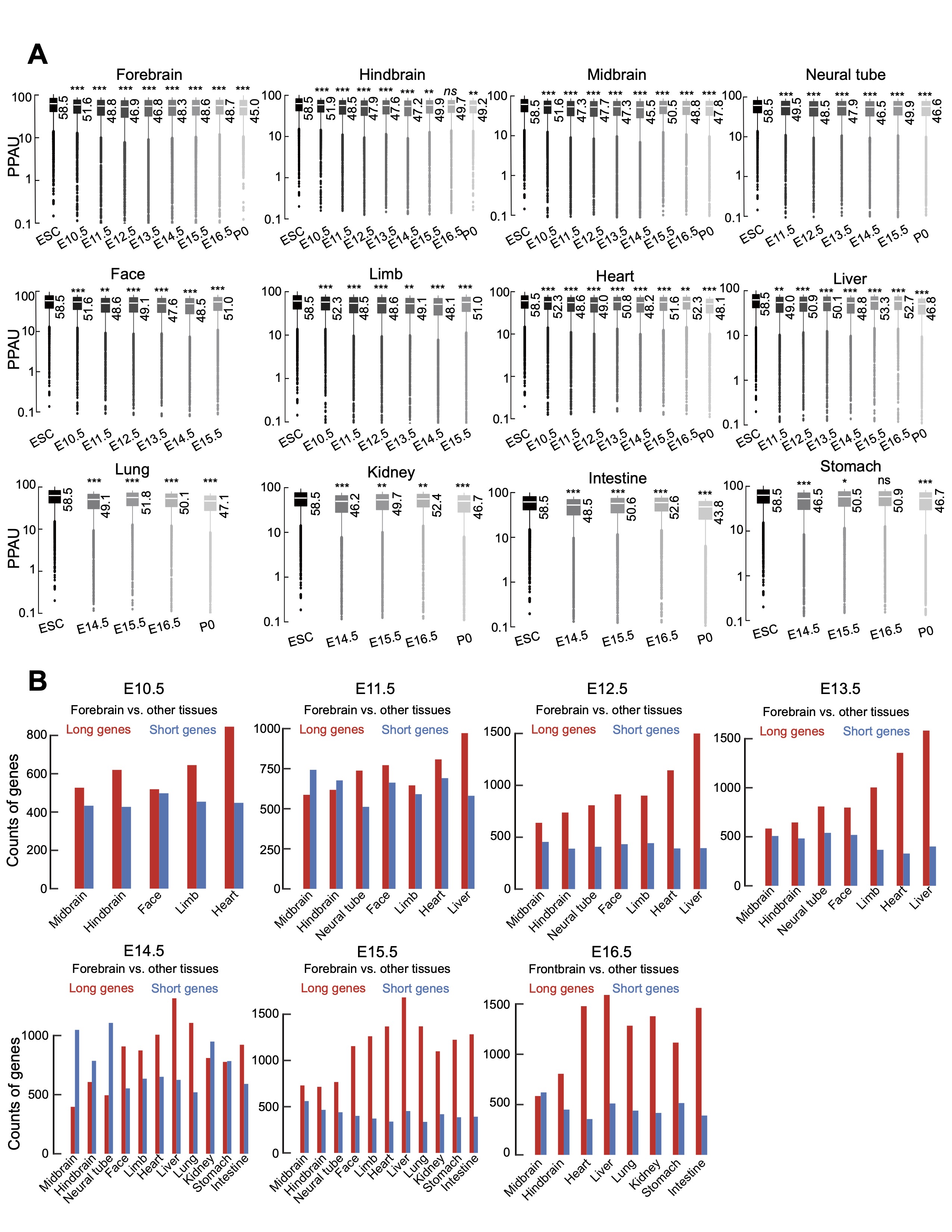


**Figure S2. Length preference of 3′ UTRs in different tissues**

A) Boxplots showing PPAUs calculated in mESC and mouse tissues (forebrain, hindbrain, midbrain, neural tube, face, limb, heart, liver, lung, kidney, intestine, and stomach) across different developmental stages. Wilcoxon rank-sum test was performed between mESC and tissues in each stage, with *, **, and *** denoting *p*-value ≤ 0.05, ≤ 0.01, and ≤ 0.001, respectively.

B) Barplots showing counts of long genes (red) and short genes (blue) by comparing forebrain and other tissues in each developmental stage.


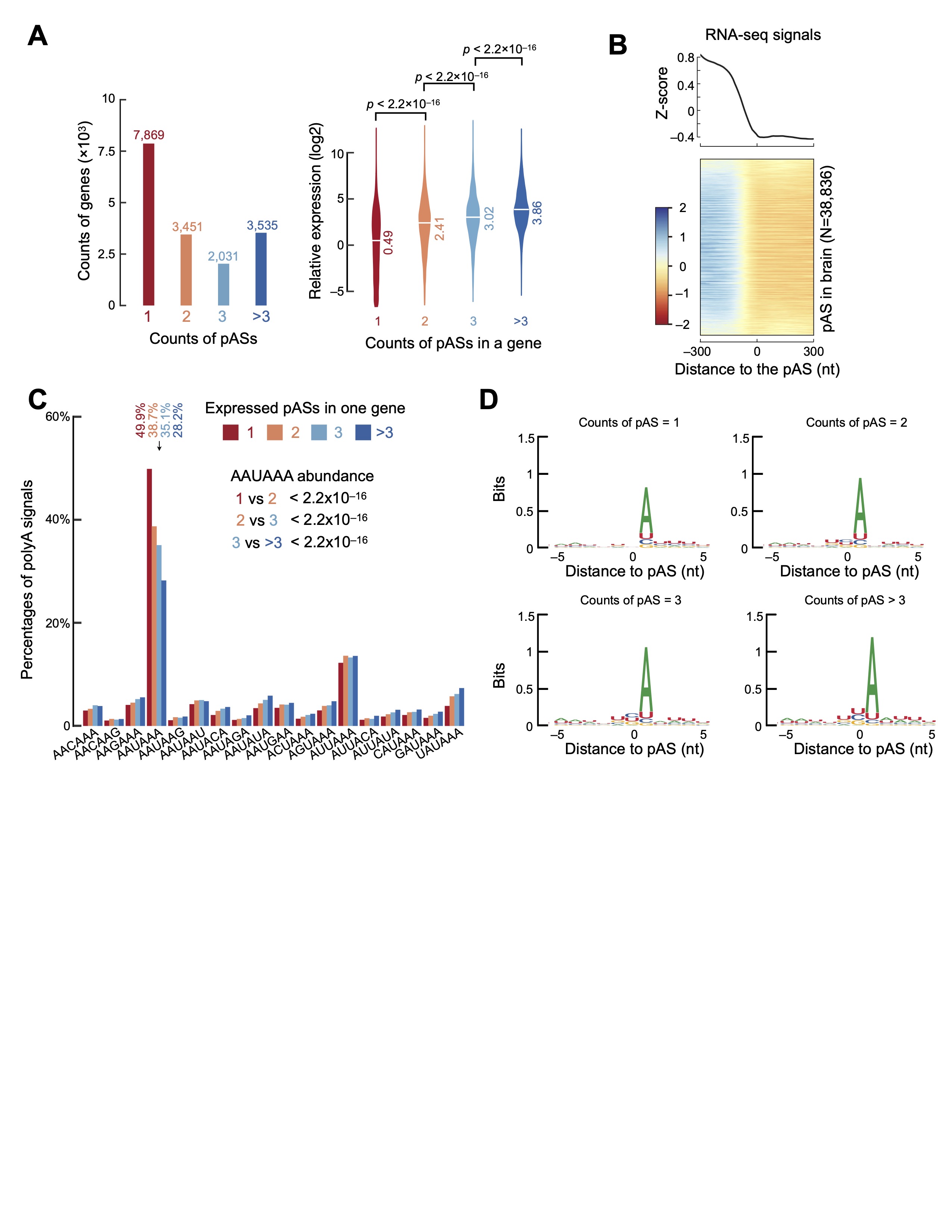


**Figure S3. Sequence context for human gene categories with different counts of expressed pASs**

A) Human genes are classified into four categories: genes with one pAS (red), two pASs (yellow), three pASs (cyan), and more than three pASs (blue), according to the number of expressed pASs in one gene (left panel). Gene categories with more expressed pASs have proportionally higher expression levels (right panel). Wilcoxon rank-sum test p-values are shown.

B) Normalized RNA-seq signal (Z-score) in the ±300-nt window centered on the 38,836 human expressed pASs in the brain tissue.

C) The percentages of 18 polyA signals in the upstream 50-nt region of expressed pASs in different gene categories. Gene categories were defined as panel A.

D) Sequence logos of expressed pASs in different gene categories. Gene categories were defined as panel A.


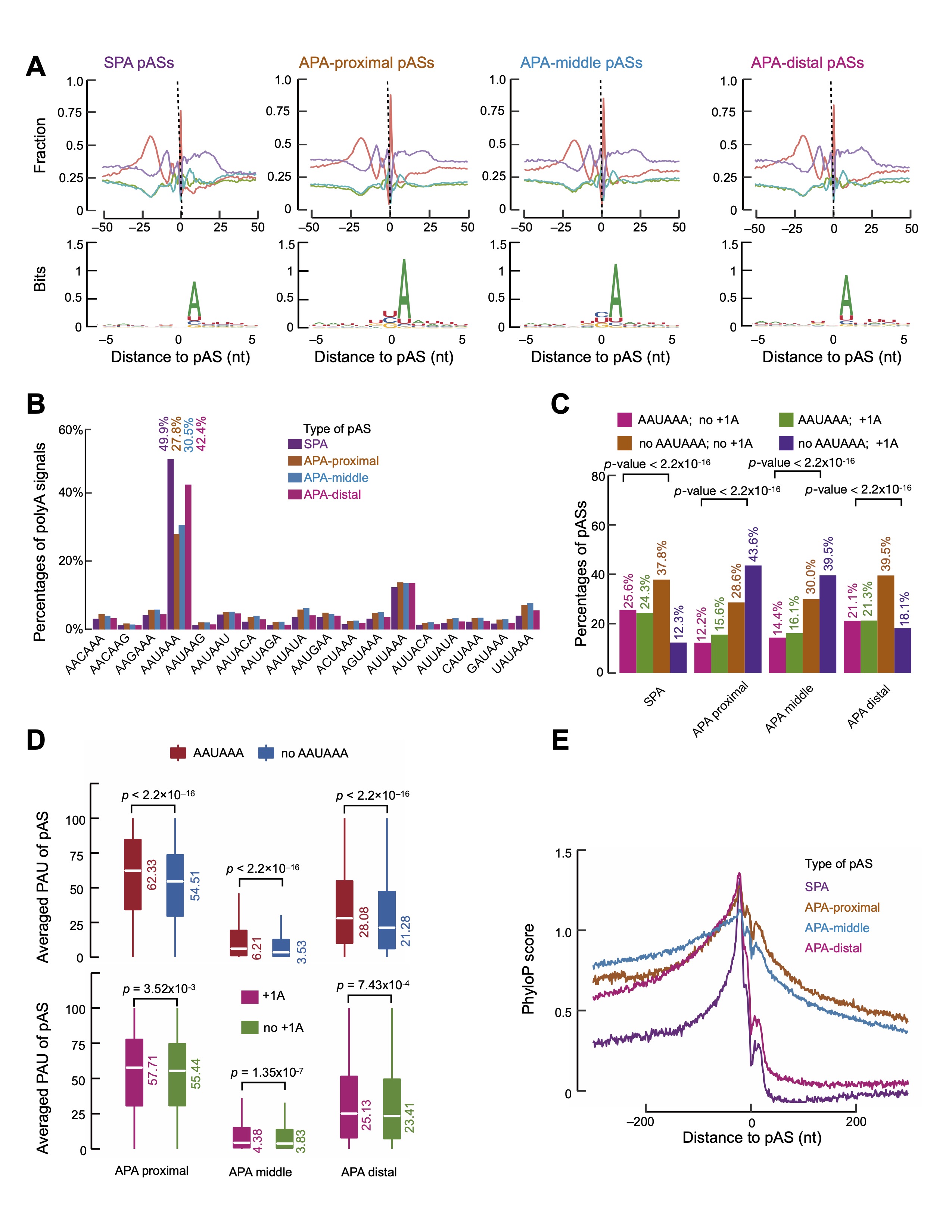


**Figure S4. Different types of human pASs exhibit distinct sequence characteristics**

A) (Top panel) nucleotide composition in the ±50-nt window centered on human SPA pASs, APA-proximal pASs, APA-middle pASs, and APA-distal pASs. (Bottom panel) sequence logos of human SPA pASs, APA-proximal pASs, APA-middle pASs, and APA-distal pASs.

B) The percentages of 18 polyA signals in the upstream 50-nt region of human SPA pASs (purple), APA-proximal pASs (yellow), APA-middle pASs (blue), and APA-distal pASs (pink).

C) Percentages of pASs which have AAUAAA in the upstream 50-nt region of pASs or adenine at the +1 position for the human SPA pASs, APA-proximal pASs, APA-middle pASs, and APA-distal pASs.

D) (Top panel) averaged PAU of pASs which have AAUAAA or not in the upstream 50-nt region for human SPA pASs, APA-proximal pASs, APA-middle pASs, and APA-distal pASs. (Bottom panel) averaged PAU of pASs which have +1A or not for human SPA pASs, APA-proximal pASs, APA-middle pASs, and APA-distal pASs. Wilcoxon rank-sum test was performed between groups and the corresponding p-values are labeled.

E) Averaged phyloP scores of the ±200-nt genomic regions centered on human SPA pASs (purple), APA-proximal pASs (yellow), APA-middle pASs (blue), and APA-distal pASs (pink).


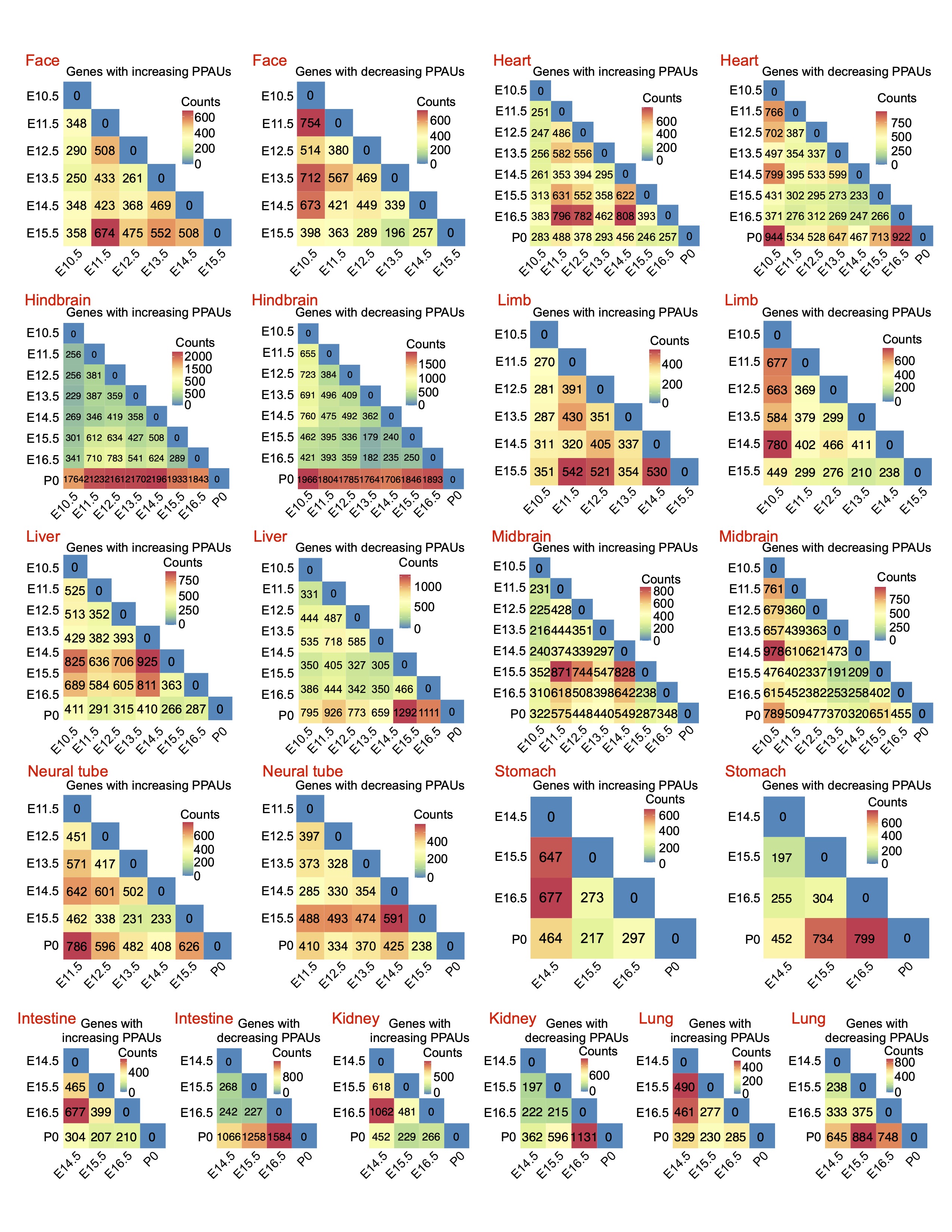


**Figure S5. Counts of genes with PPAU alteration**

Counts of genes with increasing (left panel) or decreasing (right panel) PPAUs across 11 tissues (hindbrain, midbrain, neural tube, face, limb, heart, liver, lung, kidney, intestine, and stomach) by comparing between adjacent embryonic and postnatal stages.


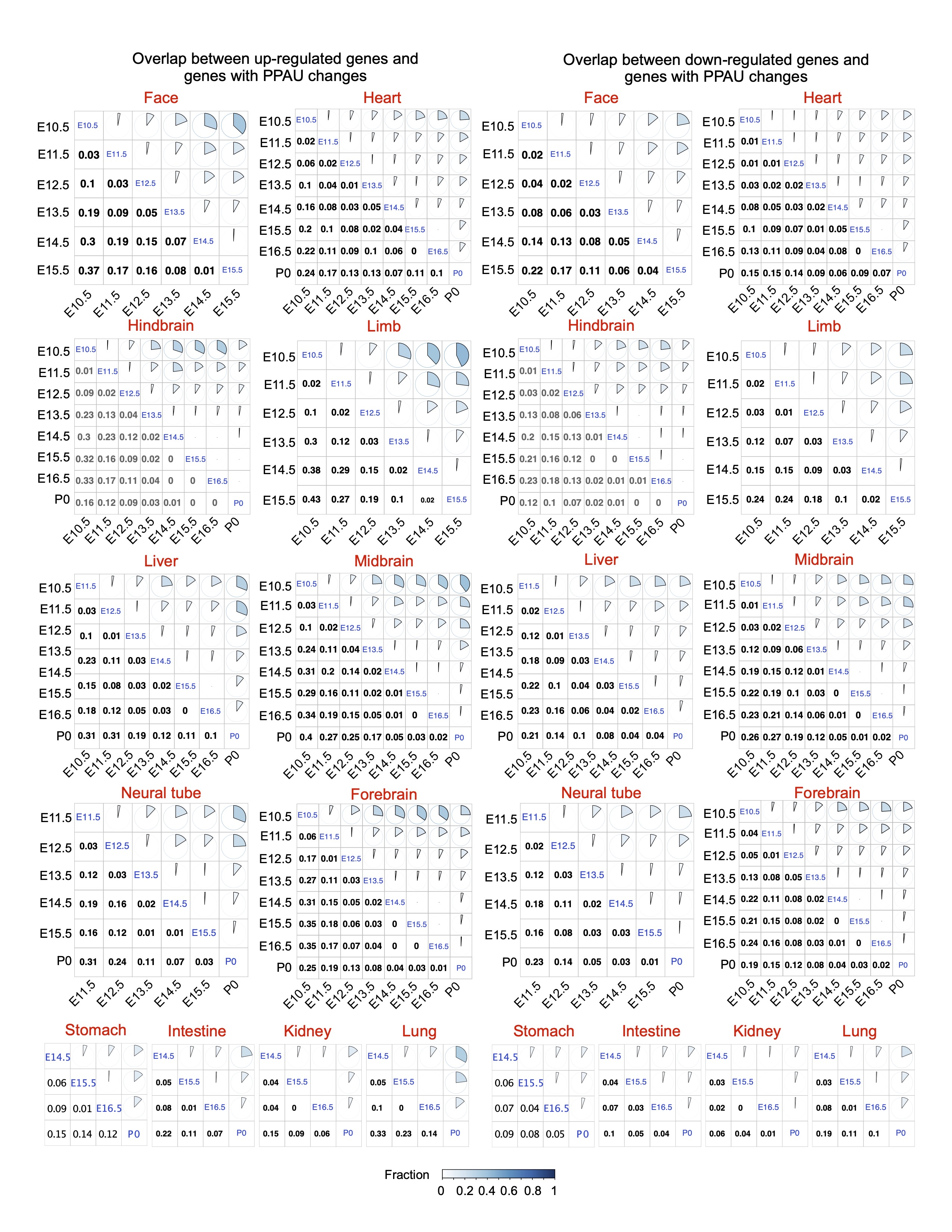


**Figure S6. Fractions of differential expressed genes overlapped with PPAU-altered genes**

Fractions of up-regulated (left panel) or down-regulated (right panel) genes overlapped with PPAU-altered genes across 12 tissues (forebrain, hindbrain, midbrain, neural tube, face, limb, heart, liver, lung, kidney, intestine, and stomach) by comparing between adjacent embryonic and postnatal stages.


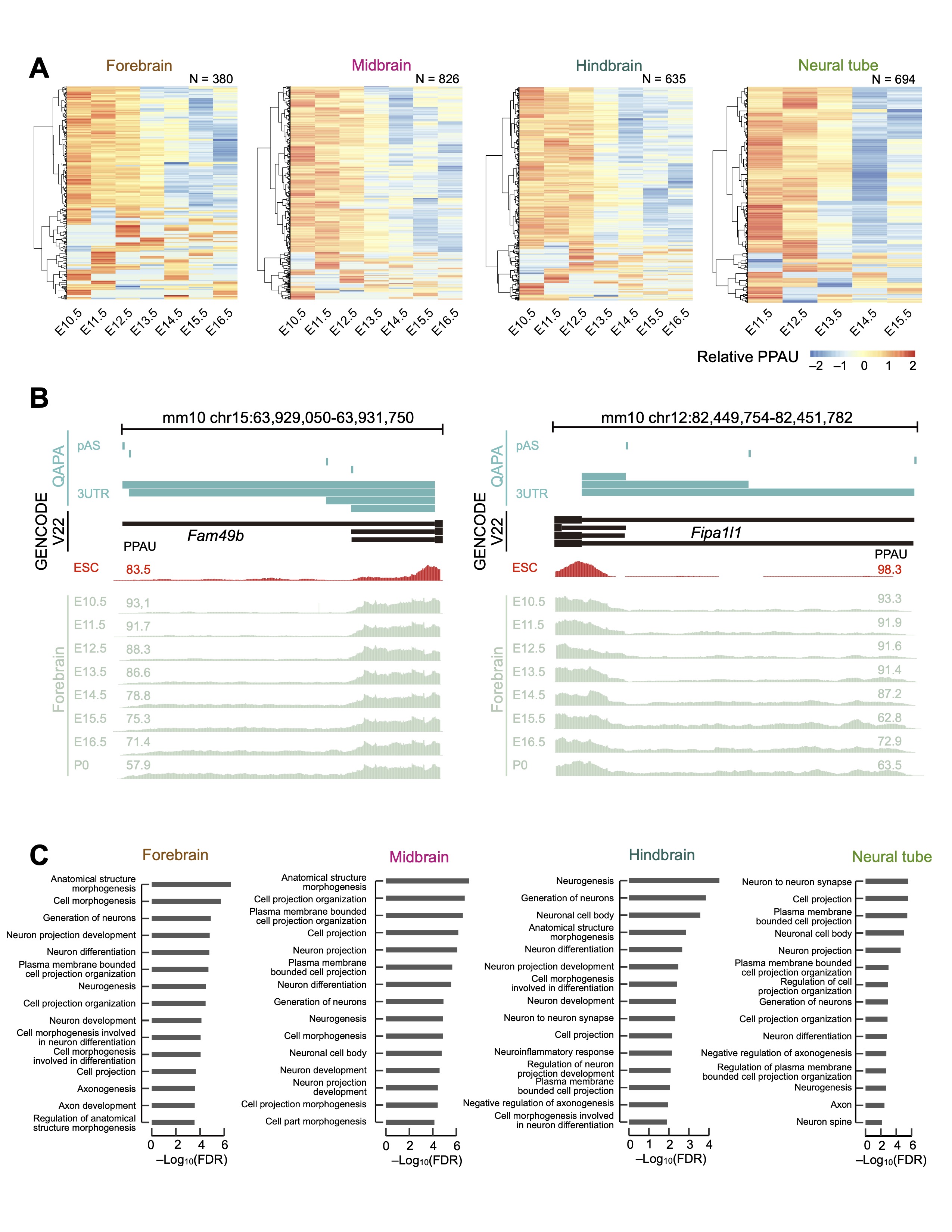


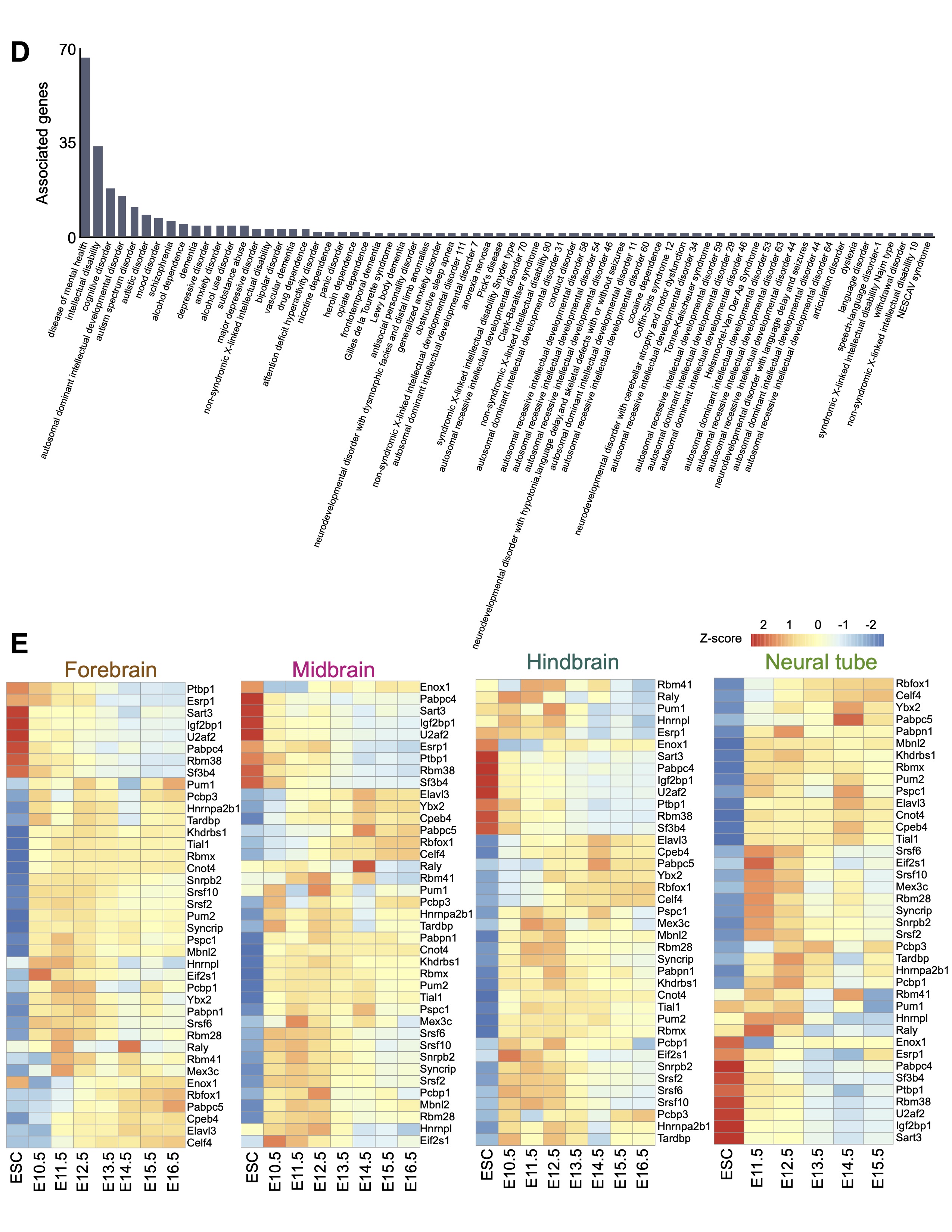


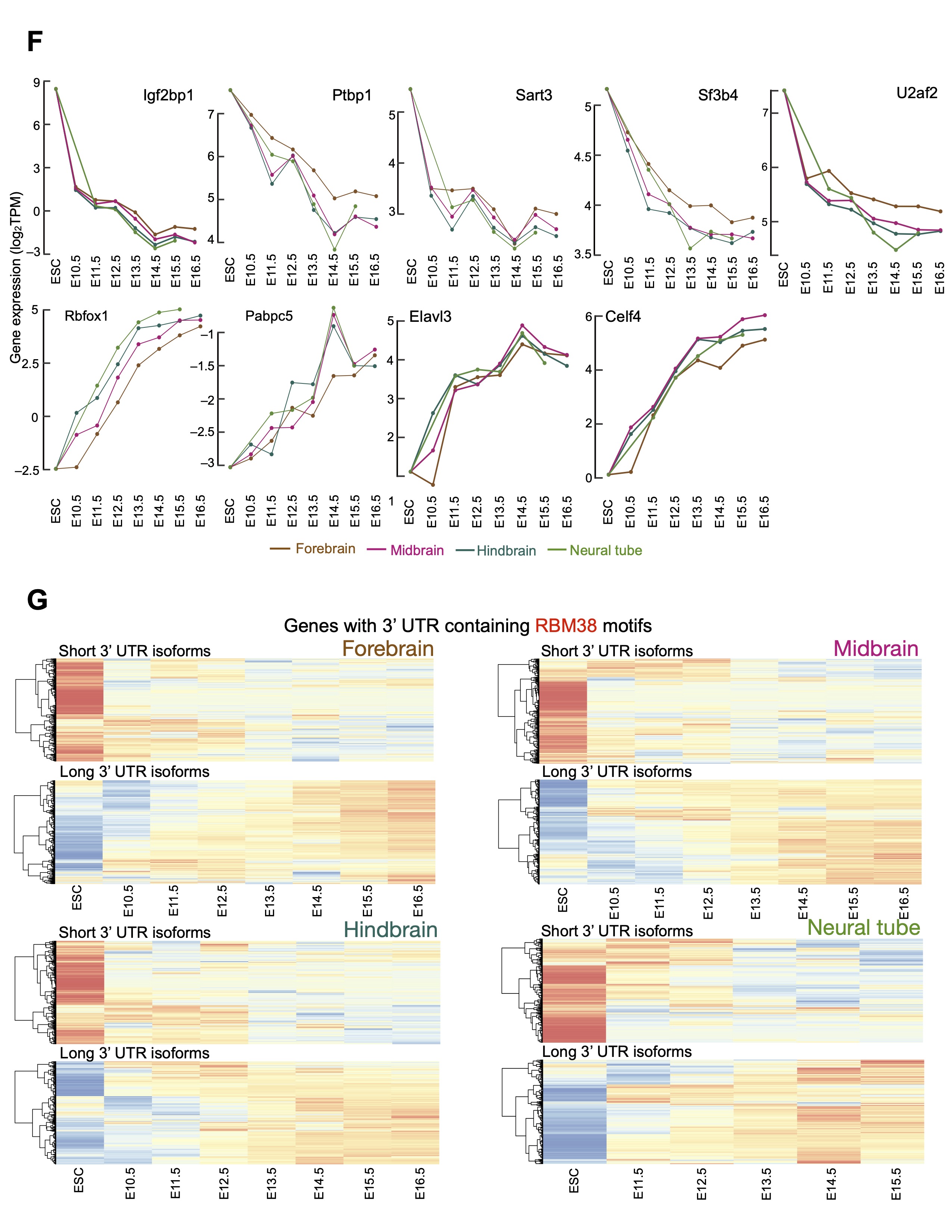


**Figure S7. Specific alternative polyadenylation patterns throughout mouse fetal brain development**

A) PPAU heatmap of genes with PPAU decreased pattern (PPAU-decreased) during mouse fetal development in four brain tissues (forebrain, midbrain, hindbrain, and neural tube).

B) APA events in genes *Fam49b* (left panel) and *Fipa1l1* (right panel) throughout mouse fetal brain development. Expressed pASs and 3′ UTR identified by QAPA are labeled on the top, and PPAUs calculated in each biosample are labeled beside the corresponding RNA-seq signal track.

C) Barplots showing top 15 GO terms enriched for PPAU-decreased genes identified in four brain tissues (forebrain, midbrain, hindbrain, and neural tube).

D) Mental health-related diseases associated with PPAU-decreased genes.

E) Relative expression of 39 identified RBPs throughout mouse fetal brain development.

F) The expression trend of nine RBPs (*Igf2bp1, Ptbp1, Sart3, Sf3b4, U2af2, Rbfox1, Pabpc5, Elavl3,* and *Celf4*) during mouse fetal brain development.

G) Relative expression of short and long isoforms in APA genes with 3′ UTR containing *Rbm38* motifs throughout mouse fetal brain development.

**
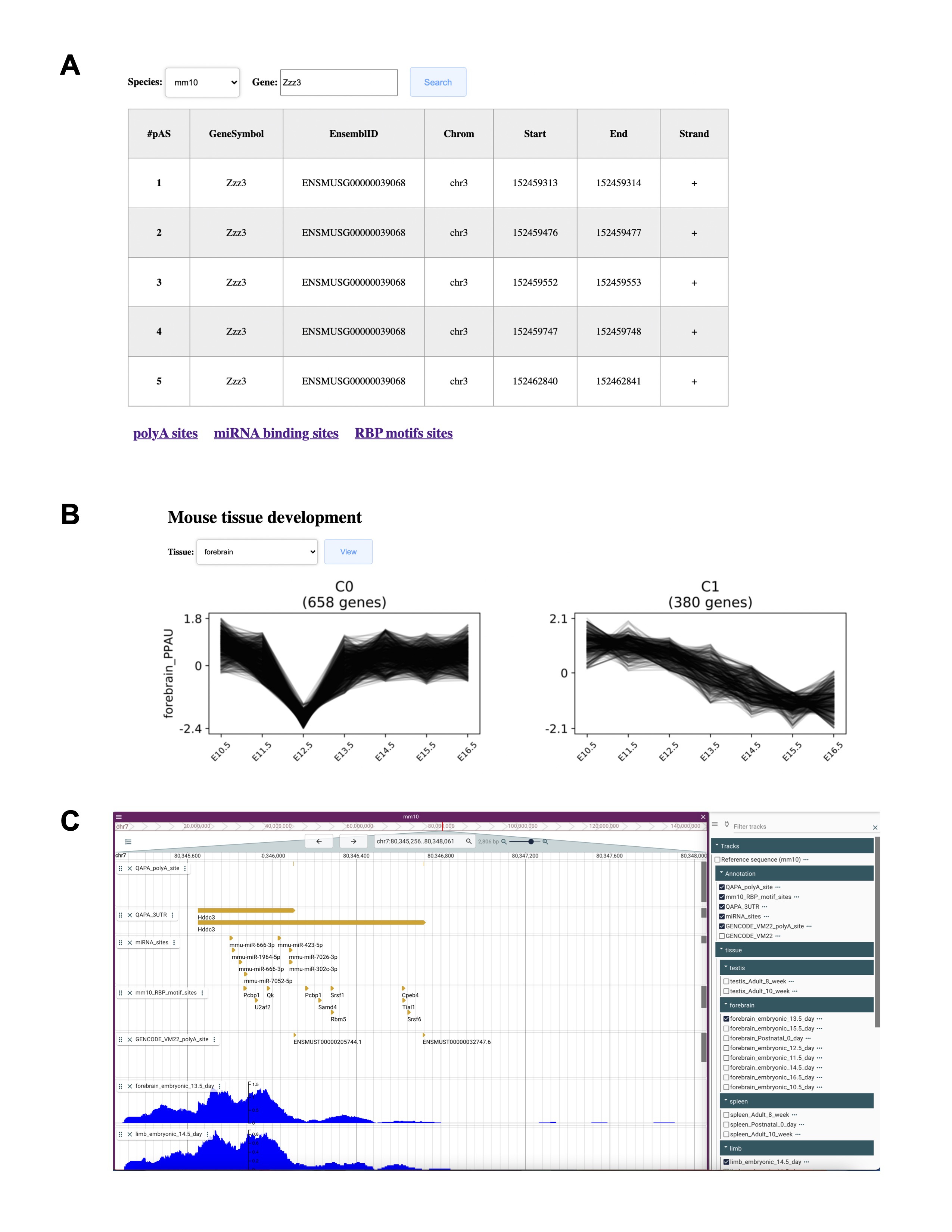
**

**Figure S8. APApedia database**

A) An example for pAS search from the search page of APApedia, which provides query support based on species, gene symbol, and Ensembl ID. APApedia will retrieve all of the pASs for this gene, together with the related miRNA binding sites as well as RBP motif sites.

B) APApedia provides the visualization for APA patterns identified throughout mouse fetal development.

C) Browser view of pASs, 3′ UTRs, and RNA-seq signals for APA events in the APApedia-embedded JBrowse.
